# N-Acetylcysteine Amide against Aβ-Induced Alzheimer’s-like Pathology in Rats

**DOI:** 10.3390/ijms241612733

**Published:** 2023-08-12

**Authors:** Ahmed Fareed Alkandari, Sampath Madhyastha, Muddanna S. Rao

**Affiliations:** Department of Anatomy, College of Medicine, Kuwait University, P.O. Box 24923, Safat 13110, Kuwait; ahmed.adbullah@grad.ku.edu.kw (A.F.A.); muddanna.rao@ku.edu.kw (M.S.R.)

**Keywords:** oxidative stress, antioxidants, N-Acetylcysteine, N-Acetylcysteine amide, hippocampus, medial prefrontal cortex

## Abstract

Oxidative stress with a depletion of glutathione is a key factor in the initiation and progression of Alzheimer’s disease (AD). N-Acetylcysteine (NAC), a glutathione precursor, provides neuroprotective effects in AD animal models. Its amide form, N-Acetylcysteine amide (NACA), has an extended bioavailability compared to NAC. This study evaluates the neuroprotective effects of NACA against Aβ1-42 peptide-induced AD-like pathology in rats. Male *Wistar* rats (2.5 months old) were divided into five groups: Normal Control (NC), Sham (SH), Aβ, Aβ + NACA and NACA + Aβ + NACA (*n* = 8 in all groups). AD-like pathology was induced by the intracerebroventricular infusion of Aβ1-42 peptide into the lateral ventricle. NACA (75 mg/kg) was administered either as a restorative (i.e., injection of NACA for 7 consecutive days after inducing AD-like pathology (Aβ + N group)), or as prophylactic (for 7 days before and 7 days after inducing the pathology (N + Aβ + N group)). Learning and memory, neurogenesis, expression of AD pathology markers, antioxidant parameters, neuroprotection, astrogliosis and microgliosis were studied in the hippocampus and the prefrontal cortex. All data were analyzed with a one-way ANOVA test followed by Bonferroni’s multiple comparison test. NACA treatment reversed the cognitive deficits and reduced oxidative stress in the hippocampus and prefrontal cortex. Western blot analysis for Tau, Synaptophysin and Aβ, as well as a histopathological evaluation through immunostaining for neurogenesis, the expression of neurofibrillary tangles, β-amyloid peptide, synaptophysin, neuronal morphology and gliosis, showed a neuroprotective effect of NACA. In conclusion, this study demonstrates the neuroprotective effects of NACA against β-amyloid induced AD-like pathology.

## 1. Introduction

Alzheimer’s disease (AD) is a neurodegenerative disorder that results in irreversible damage to the brain’s neurons. People diagnosed with AD or dementia struggle with a progressive deficit in memory, which worsens the quality of life in affected individuals as well as their caretakers. Despite the considerable research from animals to humans to combat this devastating neurodegenerative disease, no cure has been found for AD. It is hoped that a treatment will be developed that delays or at least reduces its symptoms. In terms of pathophysiology, AD patients have mainly two pathologies in their brain: the hyperphosphorylation of the Tau protein [1] and the accumulation of the β-amyloid (Aβ) protein [2]. These two pathologies, together with other factors, induce oxidative stress (OS) in the brain [3,4,5], which causes loss of synapses, neuronal transmission and eventually neuronal loss. With aging, the intracellular concentration of glutathione (GSH) decreases, leading to an overproduction of reactive oxygen species (ROS) and causing OS [6]. The OS is due to the generation of ROS allowing Tau hyperphosphorylation and Aβ accumulation. Among the major causes of OS in AD is an imbalance in radical detoxifying enzymes.

As the main intracellular thiol, GSH scavenges ROS and maintains the oxidative balance in tissues. Cysteine and GSH delivery compounds have been used to protect normal cells from redox imbalance. Therefore, thiol-containing compounds have gained special attention due to their profound role in maintaining tissue redox balance. In the past decade, a large number of natural thiols were used to combat lead-induced toxic effects. One of the most widely used thiol antioxidants has been N-Acetyl cysteine (NAC). NAC is known to exert its neuroprotective potential through two well-known mechanisms, which are its restoration of the GSH pool [7] and direct scavenging ability against ROS [8]. After entering the neuron, it rapidly hydrolyzes to yield cysteine, which can regenerate the total GSH contents and reduce excessively oxidized GSH. Preclinical data also provide evidence that NAC treatment is beneficial in AD murine models by counteracting oxidative damage [9]. NAC is known to exert neuroprotective effects on β-amyloid-induced AD-like characteristics in animal models [10,11,12,13]. There has been a recent advance in the development of a strategy to improve the pharmacokinetics of NAC. The amide form of NAC, N-Acetylcysteine amide (NACA), is said to provide extended bioavailability compared to NAC. NACA has better antioxidant and chelating properties when compared to those of NAC in vitro studies [14], and its ability to cross the blood–brain barrier was better than its parental form, NAC [15,16,17,18]. Moreover, NACA was found to be effective in many OS-related diseases. In fact, NACA was shown to be superior to the parent compound NAC in some neurotoxic cases [19,20,21]. In this way, NACA can be used at lower doses than NAC, avoiding its potential adverse effects [19]. Thus, boosting antioxidant systems, especially GSH, can reverse the cognitive loss associated with oxidative stress. The present study aimed to evaluate the neuroprotective effects of NACA against Aβ1-42 peptide-induced AD-like pathology in rats.

All forms of OS, such as lipid oxidation, increased protein oxidation levels, glycoxidation and advanced DNA oxidation, result in toxic constituents such as aldehydes, free carbonyls, ketones, peroxides, alcohols and cholestenone [22], which are features in the course of the progression of AD. The GSH has a fundamental role in the antioxidant defense system, which is in charge of the endogenous redox potential in cells [23]. It is well known that the OS has pros and cons for many pathologies of AD. For instance, there is a direct correlation between abnormal Aβ production and OS [24]. Increased lipid peroxidation and decreased vitamin E and GSH were seen in the triple transgenic mouse model of AD [25]. The initiation of OS increased Aβ plaques and Aβ1-42 peptides in another double mutant mouse model for the expression of the amyloid precursor protein [26]. It is also evident that tauopathies are also associated with OS. An experiment that involved transgenic mice carrying the human Tau P301L protein exposed the downregulation of the proteins involved in mitochondrial respiration, metabolism and antioxidant enzymes. Moreover, these mice revealed high lipid peroxidation and ROS production in the cortex, increased antioxidant enzyme activity and mitochondrial dysfunction [27]. There is no doubt that OS increases Tau phosphorylation, as demonstrated in numerous studies [28,29]. The Aβ acts as a significant trigger in the glial response since microglial and astrocytic activation are concerned with Aβ plaques [30]. Despite an early plateau in Aβ deposition in the brain, there was a constant increase in activated microglia and reactive astrocytes throughout the disease. The increased production of reactive oxygen species (ROS) was associated with age, and reduced antioxidant defense directly affected synaptic activity and neurotransmission in neurons, leading to cognitive dysfunction. Extensive studies conducted in vivo and in vitro support a direct relationship between oxidative stress and synaptic dysfunction in AD [31,32]. Hence the present study further evaluated the effect of NACA on the synaptophysin protein. Eventually all the above-mentioned pathological manifestations lead to neuronal loss. The present study also evaluated the neuronal loss in the brain regions concerned with AD pathology. The objective of the study was to evaluate the neuroprotective effect of NACA on animal models of AD using a variety of parameters which mimic human AD pathologies.

## 2. Results

### 2.1. Assessment of Spatial Learning and Memory

#### 2.1.1. Morris Water Maze—Learning

**Figure 1:**
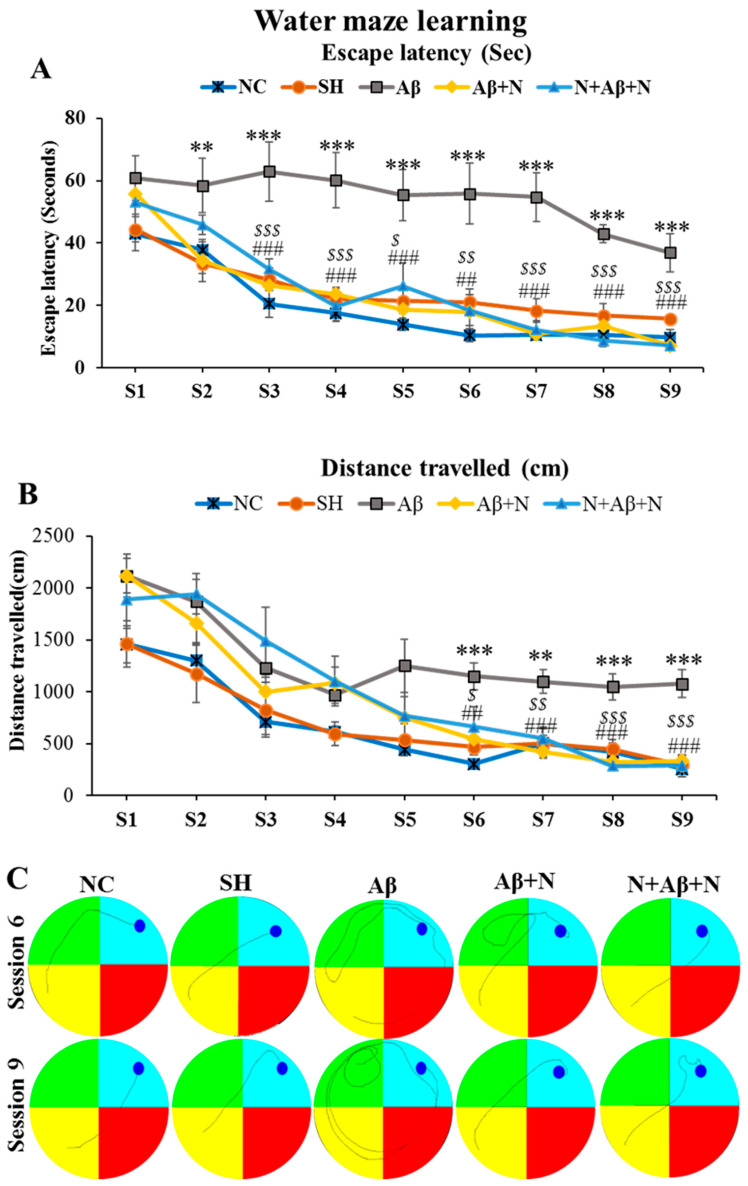
Performance of rats in the various groups during Morris water maze learning sessions. (**A**) Escape latency, (**B**) Distance travelled. Note that the rats infused with Aβ1-42 peptide into the lateral ventricle (the Aβ group) had significantly greater escape latency in sessions 2–9, and they travelled a longer distance before reaching the platform in sessions 5–9. NC or SH vs. Aβ, *** *p* < 0.001 and ** *p* < 0.01; Aβ vs. N + Aβ, ^###^ *p* < 0.001 and ^##^ *p* < 0.01; Aβ vs. N + Aβ + N, ^$$$^ *p* < 0.001, ^$$^ *p* < 0.01 and ^$^ *p* < 0.05 (*n* = 18 in all groups). (**C**) Representative video tracking of the rats in the various groups during Morris water maze learning sessions 6 and 9. Dark blue circle indicates the position of the circular platform submerged 1cm below the water level in the platform quadrant (light blue). Note that rats in NC and SH groups were able to learn and reach the hidden platform with a shortest path, while rats in the Aβ group took longer path to reach the platform, even in the 9th session, suggesting a learning deficit. On the other hand, Aβ1-42 peptide-infused rats that received post-treatment and pre- and post-treatment with NACA in the Aβ + N and N + Aβ + N groups, respectively, reached the hidden platform with the same path as the NC and SH rats, denoting intact learning.

*A. Escape latency:* During the learning sessions, rats infused with Aβ1-42 peptide into the lateral ventricle (the Aβ group) had significantly greater escape latency in sessions 2–9 compared to the NC and SH groups (*p* < 0.01–0.001) (Figure 1A), indicating a learning deficit in them. There was a significantly decreased escape latency in animals that received post-treatment or pre- and post-treatment with NACA in the Aβ + N and N + Aβ + N groups compared to the Aβ group in sessions 3—9 (Aβ vs. Aβ + N, *p* < 0.01–0.001; Aβ vs. N + Aβ + N, *p* < 0.01–0.00) (Figure 1A). No significant difference was found in escape latency among (i) the NC and SH groups, (ii) the NC/SH and Aβ + N or N + Aβ + N groups and (iii) the Aβ + N and N + Aβ + N groups in all sessions (Figure 1A).

*B. Distance travelled to reach the platform:* Throughout the learning sessions, animals infused with Aβ1-42 peptide into the lateral ventricles (the Aβ group) travelled a longer distance before reaching the platform in sessions 6–9 compared to the NC and SH groups (*p* < 0.01–0.001) (Figure 1B), indicating a learning deficit in them. There was a significantly decreased distance travelled in animals that received post-treatment or pre- and post-treatment with NACA in the Aβ + N and N + Aβ + N groups compared to the Aβ group in sessions 6–9 (Aβ vs. Aβ + N, *p* < 0.01–0.001; Aβ vs. N + Aβ + N, *p* < 0.01–0.001) (Figure 1B). No significant difference was found in distance travelled among (i) the NC and SH groups, (ii) the NC/SH and Aβ + N or N + Aβ + N groups and (iii) the Aβ + N and N + Aβ + N groups in all sessions (Figure 1B). Video tracking of the animals in the various groups during learning sessions 6 and 9 of the Morris water maze test is shown in Figure 1C.

#### 2.1.2. Morris Water Maze—Memory Retention

**Figure 2:**
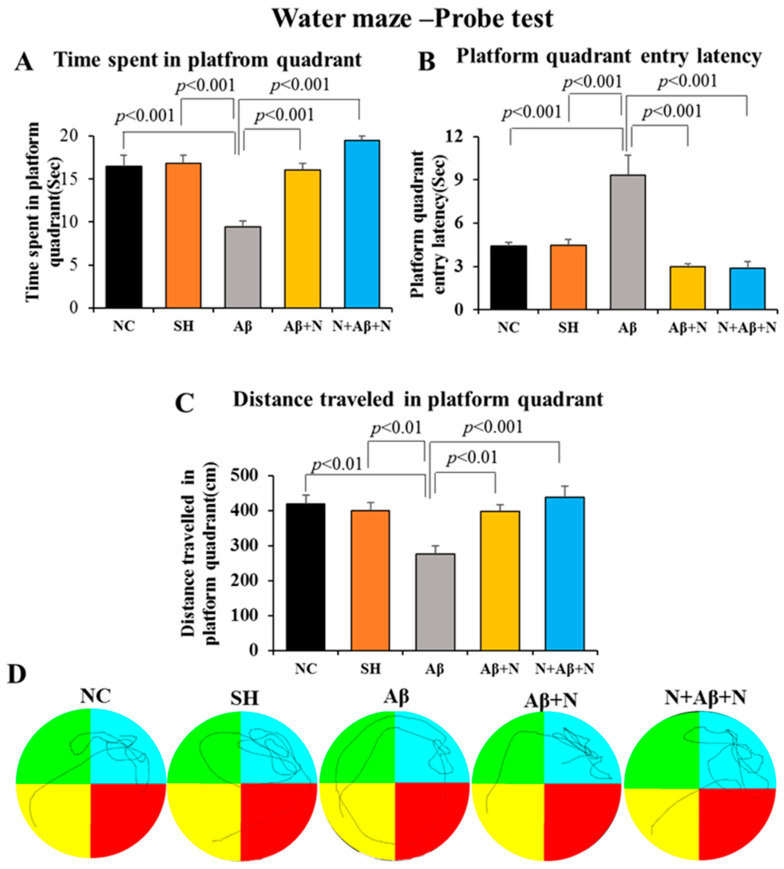
Performance of the rats in the various groups during the Morris water maze memory retention test. (**A**) Time spent in the platform quadrant. (**B**) Platform quadrant entry latency. (**C**) Distance travelled in the platform quadrant. Note that rats in the Aβ group had a long latency to enter the platform quadrant, spent less time there and travelled less distance compared to the NC or SH groups, indicating a memory deficit in them. However, the Aβ + N and N + Aβ + N groups showed a significant improvement in the same parameters compared to the Aβ group (*n* = 18 in all groups). (**D**) Representative video tracking of a rat in the various groups during Morris water maze memory retention test. Note that the rats in NC and SH groups were able to learn the location of hidden platform while rats in the Aβ group did not, suggesting a memory deficit. However, rats in the Aβ + N and N + Aβ + N groups did remember the quadrant where the hidden platform was present, denoting intact memory.

*A. Time spent in the platform quadrant:* Rats infused with Aβ1-42 peptide into the lateral ventricles (the Aβ group) stayed for a significantly shorter duration (9.4 s) in the platform quadrant compared to that of the NC and SH groups (16.4 and 16 s, respectively, *p* < 0.001) during the memory retention test (Figure 2A). In contrast, rats in the Aβ + N and N + Aβ + N groups stayed for significantly longer (16.0 and 19.5 s, respectively, *p* < 0.001) in the platform quadrant in comparison to the Aβ group (9.5 s). No significant difference was found in the time spent in the platform quadrant during the memory retention test among (i) the NC and SH groups, (ii) the NC/SH and Aβ + N or N + Aβ + N groups and (iii) the Aβ + N and N + Aβ + N groups (Figure 2A).

*B. Latency to reach the platform quadrant:* Rats infused with Aβ1-42 peptide into the lateral ventricles (the Aβ group) took a significantly longer time(9.3 s) to reach the hidden platform in the platform quadrant compared to that of the NC and SH groups (4.4 and 4.4 s, respectively, *p* < 0.01) (Figure 2B). In contrast, rats in the Aβ + N and N + Aβ + N groups took significant less time (2.9 and 2.8 s, respectively, *p* < 0.001) to reach the platform quadrant compared to that of the Aβ group (Figure 2B). No significant difference was found in latency to reach the platform quadrant during the memory retention test among (i) the NC and SH groups, (ii) the NC/SH and Aβ + N or N + Aβ + N groups and (iii) the Aβ + N and N + Aβ + N groups (Figure 2B).

*C. Distance traveled in the platform quadrant:* Rats infused with Aβ1-42 peptide into the lateral ventricles (the Aβ group) traveled a significantly shorter distance (275 cm) in the platform quadrant in comparison to the NC and SH groups (418 and 400 cm, respectively, *p* < 0.001) (Figure 2C). In contrast, rats in the Aβ + N and N + Aβ + N groups traveled a significant longer distance (397 and 436 cm, respectively, *p* < 0.001) in the platform quadrant compared to the Aβ group (275 cm). No significant difference was found in the distance travelled in the platform quadrant during the memory retention test among (i) the NC and SH groups, (ii) the NC/SH and Aβ + N or N + Aβ + N groups and (iii) the Aβ + N and N + Aβ + N groups (Figure 2C).

Figure 2D illustrates the video tracking of the animals in the various groups during the probe test. Note that during the probe test, rats infused with Aβ1-42 peptide into the lateral ventricle (the Aβ group) took a longer path to reach the hidden platform compared to the rest of the groups, indicating a learning deficit. On the other hand, the Aβ1-42 peptide-infused rats that received post-treatment and pre- and post-treatment with NACA in the Aβ + N and N + Aβ + N groups reached the hidden platform with the shortest route and spent their time in the platform quadrant, searching for the hidden platform.

### 2.2. Assessment of Passive Avoidance Learning and Memory Using Passive Avoidance Test

#### 2.2.1. Passive Avoidance—Learning

**Figure 3:**
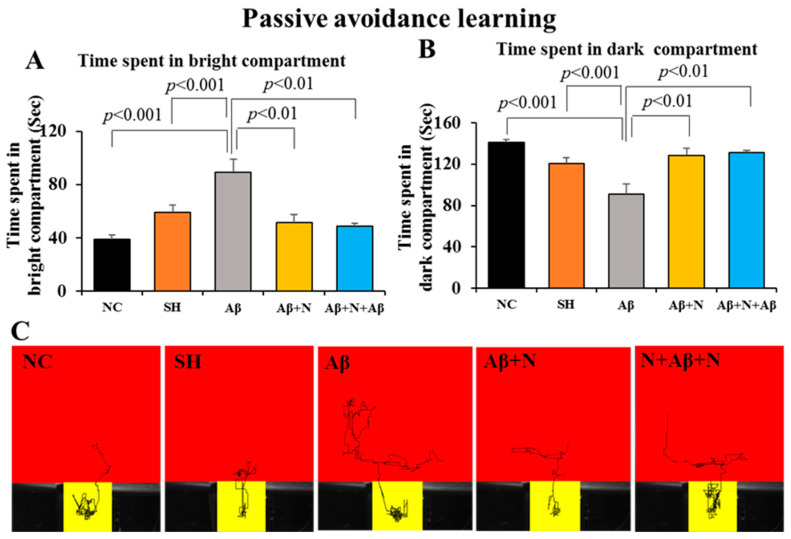
Mean time spent in the bright (**A**) and dark (**B**) compartments by the rats in the various groups during passive avoidance learning sessions. Note that the Aβ group of rats spent significantly more time in the bright compartment (conversely less time in dark compartment) in comparison to NC and SH groups. Time spent in the bright compartment was significantly diminished in Aβ + N and N + Aβ + N groups (conversely increased in the dark compartment) compared to the Aβ group (*n* = 18 in all groups). (**C**) Representative video tracking of a rat in various groups during the last (3rd) learning trial of the passive avoidance test. Note that rats in NC and SH groups explored the dark compartment for longer than the bright compartment, while rats in the Aβ group explored both compartments. Rats in the Aβ + N and N + Aβ + N groups spent significantly longer in the dark compartment in comparison to the bright compartment like NC or SH groups.

*A. Time spent in the bright compartment:* Rats infused with Aβ1-42 peptide into the lateral ventricle (the Aβ group) spent a significantly longer time in the bright compartment (89.0 s) in comparison to the NC and SH groups (38.8 and 59.0 s, respectively, *p* < 0.001) during the learning sessions. Whereas Aβ1-42 peptide-infused rats that received post-treatment and pre- and post-treatment with NACA (Aβ + N and N + Aβ + N groups respectively) spent a significantly shorter time (51.3 and 48.6 s, respectively, *p* < 0.001) in the bright compartment in comparison to the Aβ group (Figure 3A). No significant difference was found in the time spent in the bright compartment among (i) the NC and SH groups, (ii) the NC/SH and Aβ + N or N + Aβ + N groups, (iii) and the Aβ + N and N + Aβ + N groups.

*B. Time spent in the dark compartment:* Rats infused with Aβ1-42 peptide into lateral ventricles in the Aβ group spent significantly less time in the dark compartment (90.9 s) in comparison to the NC and SH groups (141.1 and 120.9 s, respectively, *p* < 0.01). Whereas rats that received post-treatment and pre- and post-treatment with NACA in the Aβ + N and N + Aβ + N groups spent significantly more time (128.7 and 131.1 s, respectively, *p* < 0.01) in the dark compartment in comparison to the Aβ group (Figure 3B).

Thus, during the 180 sec of the learning trials, rats infused with Aβ1-42 peptide into the lateral ventricle (the Aβ group) searched the two compartments, whereas the NC and SH groups spent more time in the dark compartment, indicating a deficit in exploratory learning behavior in the Aβ group. On the other hand, the Aβ1-42 peptide-infused rats that received post-treatment and pre- and post-treatment with NACA (Aβ + N and N + Aβ + N groups) stayed for a shorter period in the bright compartment and a longer period in the dark compartment during the learning session compared to the Aβ group, denoting an intact exploratory learning behavior in them. The video tracking of the animals in the various groups during the last (3rd) learning trial in the passive avoidance test is shown in Figure 3C.

#### 2.2.2. Passive Avoidance—Memory Retention

**Figure 4:**
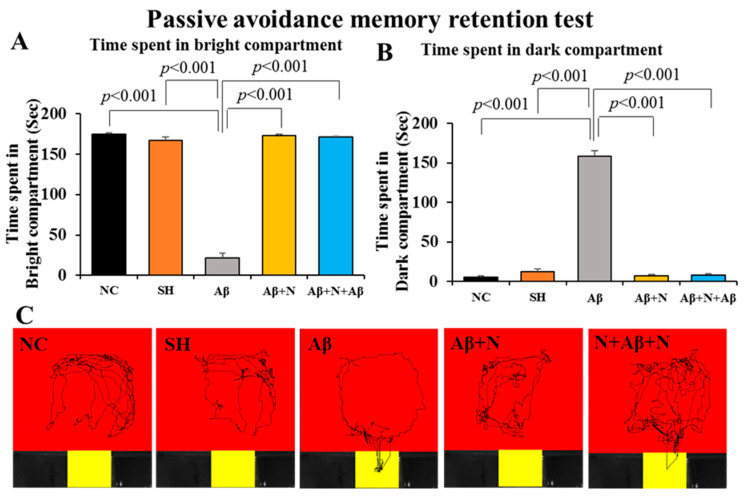
Time spent in the bright and dark compartments during passive avoidance memory retention test. (**A**) Bright compartment, and (**B**) dark compartment. Note that rats in the Aβ group stayed for significantly less time in the bright compartment (i.e., more time in dark compartment) in comparison to the NC or SH groups, suggesting a memory impairment. The Aβ + N and N + Aβ + N groups stayed significantly longer in the bright compartment (i.e., less time in dark compartment) in comparison to the Aβ group, suggesting a good memory (*n* = 18 in all groups). (**C**) Representative video tracking of a rat in the various groups during passive avoidance memory retention test. Note that rats in NC or SH groups avoided entering the dark compartment, in which a foot shock was previously delivered, while rats in the Aβ group did enter the dark compartment, suggesting a memory deficit. However, rats in Aβ + N and N + Aβ + N groups did avoid entering the dark compartment, like control groups, denoting intact memory.

*A. Time spent in the bright compartment:* Rats infused with Aβ1-42 peptide into the lateral ventricles (the Aβ group) stayed for significantly less time in the bright compartment (21.1 s) in comparison to NC and SH groups (174.5 and 167.4 s, respectively, *p* < 0.001) (Figure 4A). Whereas Aβ1-42 peptide-infused rats that received post-treatment and pre- and post-treatment with NACA (Aβ + N and N + Aβ + N groups) spent a significantly longer time (172.8 s in Aβ + N group, *p* < 0.001 and 171.3 s, in N + Aβ + N group, *p* < 0.001) in the bright compartment in comparison to the Aβ group (Figure 4A).

*B. Time spent in the dark compartment:* Rats infused with Aβ1-42 peptide into the lateral ventricle the Aβ group stayed significantly longer in the dark compartment (158.8 s) in comparison to NC and SH groups (5.8 and 12.5 s, respectively, *p* < 0.001). Whereas Aβ1-42 peptide-infused rats that received post-treatment and pre- and post-treatment with NACA (Aβ + N and N + Aβ + N) groups spent significantly less time (7.1 s, in Aβ + N group *p* < 0.001; and 8.6 s, in N + Aβ + N group, *p* < 0.001) in the dark compartment in comparison to the Aβ group (Figure 4B).

Thus, during the 180 s. period of the memory retention session, rats infused with Aβ1-42 peptide into the lateral ventricle (the Aβ group) stayed less time in the bright and longer in the dark compartment in comparison to those in NC/SH groups, indicating a memory deficit. On the other hand, rats that received post-treatment and pre- and post-treatment with NACA in the Aβ + N and N + Aβ + N groups, stayed for a longer period in the bright compartment and a shorter period in the dark compartment than those in the Aβ group during the memory retention test denoting intact avoidance memory. Figure 4C illustrates the video tracking of the animals in the various groups.

### 2.3. Results of the Biochemical and Morphological Studies

#### 2.3.1. Doublecortin Protein Expression in the Hippocampus (Neurogenesis)

**Figure 5:**
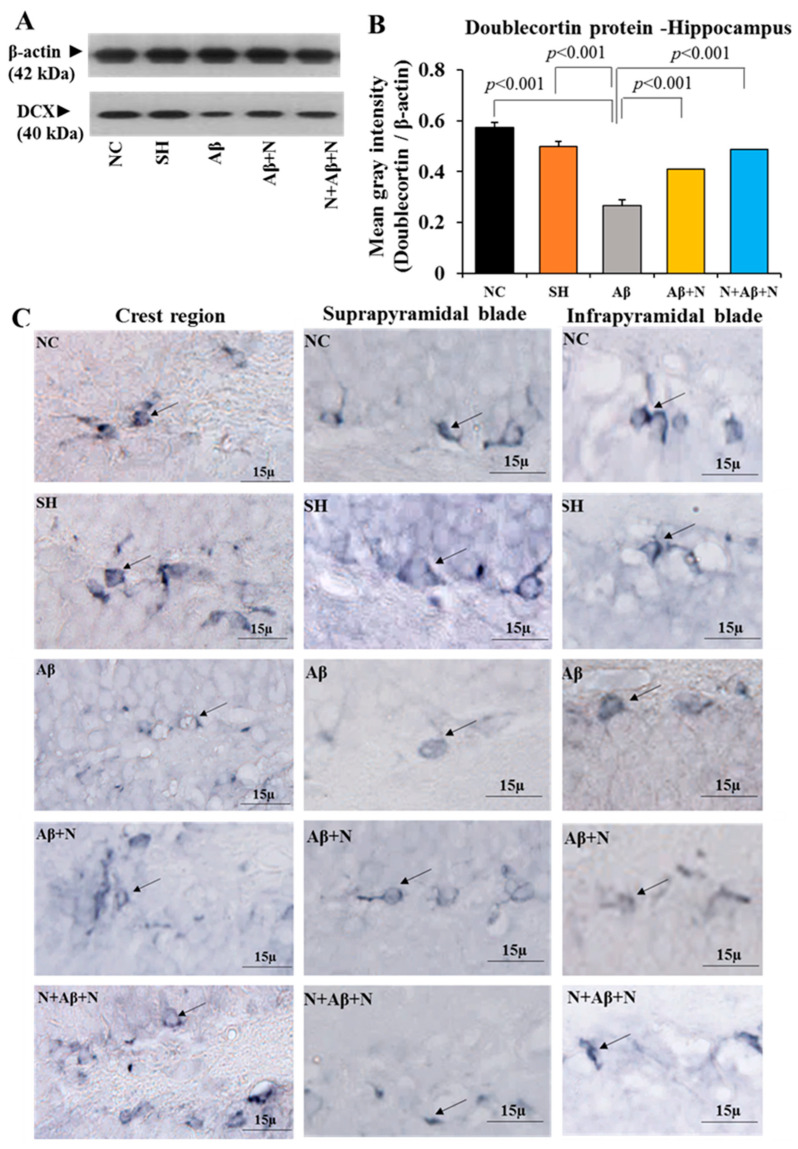
Doublecortin (DCX) protein content in the hippocampus tissue in the different groups. (**A**) Immunoblot of DCX. (**B**) Mean gray intensity (DCX/β-actin) of the immunoblots (Mean ± SEM). Note the significantly reduced DCX content in the Aβ groups when compared to NC or SH groups indicates a decreased neurogenesis. DCX content is significantly increased in Aβ + N and N + Aβ + N groups compared to Aβ, even though it does not reach the NC level (*n* = 6 in all groups). (**C**) Photomicrographs of doublecortin immunopositive neurons (arrows) in the crest, suprapyramidal blade and infrapyramidal blade regions of the dentate gyrus in the various groups. Note the diminished DCX-positive, newly born neurons (neurogenesis) in the Aβ group in comparison to NC and SH groups, and the increased neurogenesis in Aβ + N and N + Aβ + N groups compared to the Aβ group.

The DCX protein content in the rat hippocampal tissue was significantly decreased in the hippocampal tissue of the rats in the Aβ group when compared to both the NC and SH groups of rats (NC/SH vs. Aβ, *p* < 0.001). Treatment with NACA in Aβ1-42 peptide-infused rats showed a significantly increased DCX content in the hippocampal tissue compared to in the Aβ group (Aβ vs. Aβ + N, *p* < 0.001; Aβ vs. N + Aβ + N, *p* < 0.001) (Figure 5A,B). However, this increase did not reach the control level in the Aβ + N group (NC vs. Aβ + N, *p* < 0.05). No significant difference was found in the DCX content among the Aβ + N and N + Aβ + N groups and between the NC and SH groups (Figure 5A,B).

#### 2.3.2. Doublecortin Immunostaining 

The immunostaining of the brain sections with anti-DCX antibody to stain the newly generated neurons in the dentate gyrus region showed a smaller numerical density of DCX-positive neurons in the Aβ group in the crest region, infrapyramidal blade and suprapyramidal blades (Figure 5C) of the dentate gyrus compared to the NC and SH groups. The numerical density of DCX-positive new neurons in all the above regions was increased in both the Aβ + N and N + Aβ + N groups, noticeably more in the N + Aβ + N group. The new neurons in the Aβ + N and N + Aβ + N groups seem to be normal, with mature neuronal features such as vertically oriented dendrites. The increase in DCX-positive neurons indicates enhanced neurogenesis in the NACA-treated groups.

A Western blot analysis of the DCX protein content and immunostaining for the DCX-positive neurons in the dentate gyrus together suggest that NACA enhances the neurogenesis in the dentate gyrus region of the hippocampus in animals infused with Aβ1-42 peptide.

### 2.4. Synaptophysin Protein Expression in the Hippocampus and Prefrontal Cortex

#### 2.4.1. Synaptophysin Protein Level in the Hippocampus

**Figure 6:**
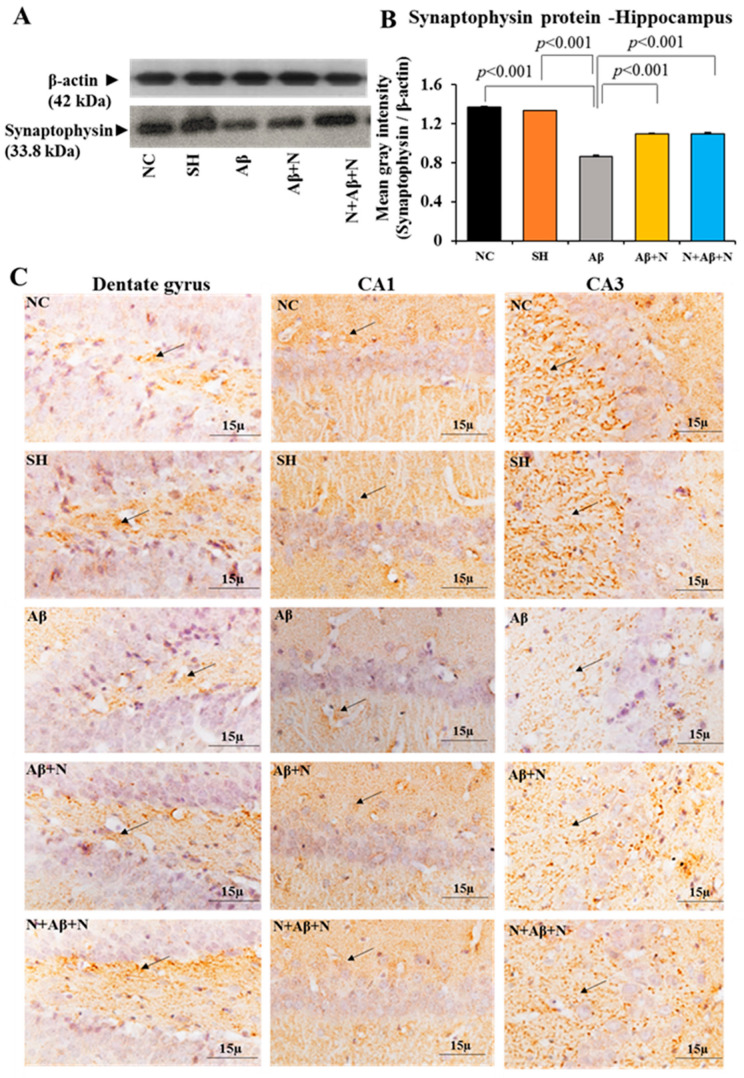
Synaptophysin (SYN) protein content in the hippocampus in the various groups. (**A**) Immunoblot of SYN. (**B**) Mean gray intensity (SYN/β-actin) of the immunoblots (Mean ± *SEM*). Note the significantly reduced SYN content in the Aβ group in comparison to the NC and SH groups, indicating fewer synapses. SYN content is significantly increased in the Aβ + N and N + Aβ + N groups compared to the Aβ group, even though it has not reached the NC level (*n* = 6 in all groups). (**C**) Photomicrographs of the dentate gyrus, CA1 and CA3 regions of the hippocampus showing the expression of synaptophysin (arrows) in various groups. Note the decreased expression of SYN (arrows) in the Aβ group compared to the NC and SH groups, and the increased expression in the Aβ + N and N + Aβ + N groups compared to the Aβ group in all regions.

SYN protein expression had a significant decrease in the hippocampus of the Aβ group in comparison to both the NC and SH groups of animals (*p* < 0.001) (Figure 6A,B). NACA treatment (both pre and post icv Aβ1-42 peptide infusion), elevated the SYN expression significantly compared to the Aβ group (Aβ vs. Aβ + N, *p* < 0.001; Aβ vs. N + Aβ + N, *p* < 0.001). However, this elevation did not reach the level in control groups (NC or SH). No significant (*p* > 0.05) difference was found in expression of SYN protein among the NC and SH or Aβ + N and N + Aβ + N groups (Figure 6A,B).

#### 2.4.2. Synaptophysin Immunostaining in the Hippocampus

Rat brain sections immunostained with an anti-SYN antibody showed positive immunostaining in all regions of the hippocampus. The expression of SYN in the dentate gyrus crest, CA1 and CA3 (Figure 6C), dentate hilus, supra- and infrapyramidal blade regions (Supplementary Figure S1) in the Aβ group was less compared to all the other groups, indicating fewer synapses in all regions in them. The expression of SYN in the NACA-treated groups (Aβ + N and N + Aβ + N) was higher than in Aβ1-42 peptide-infused group in all the regions studied (as stated above), which indicates improved synapses in all the regions studied. These findings indicate that SYN expression in the hippocampus was severely affected after the icv infusion of Aβ1-42 peptide (AD model). Both post- and pre- and post-NACA treatment in the Aβ group reversed these deficits by increasing the expression of SYN. However, they failed to normalize the deficits to the control level.

#### 2.4.3. Synaptophysin Protein Estimation in the Medial Prefrontal Cortex

**Figure 7:**
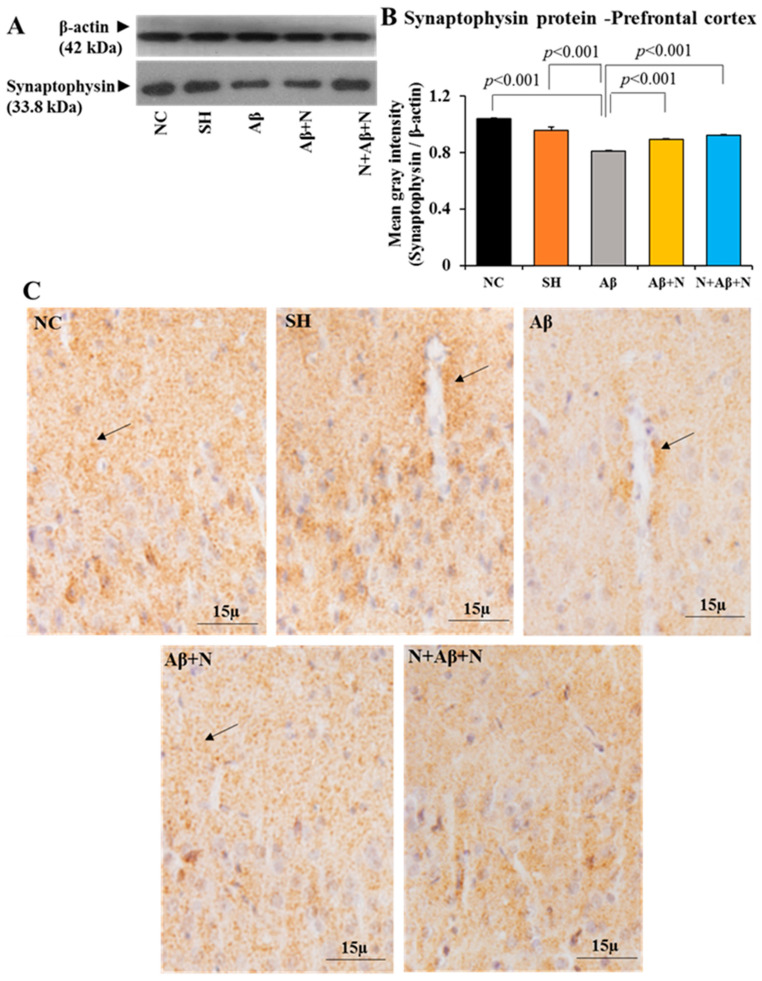
Synaptophysin (SYN) protein content in the medial prefrontal cortex in the various groups. (**A**) Immunoblot of SYN. (**B**) Mean gray intensity (SYN/β-actin) of the immunoblots (Mean ± SEM). Note the significantly diminished SYN content in the Aβ group in comparison to the NC and SH groups, indicating fewer synapses. SYN content is significantly increased in Aβ + N and N + Aβ + N groups compared to the Aβ group, even though it does not reach the NC level (*n* = 6 in all groups). (**C**) Photomicrographs of medial prefrontal cortex showing the expression of synaptophysin(arrows) in various groups. Note the diminished SYN expression in the Aβ group in comparison to the NC and SH groups, and the increased expression in Aβ + N and N + Aβ + N groups compared to the Aβ group.

There was a significant reduction of SYN protein content in the mPFC of the Aβ group in comparison to both the NC and SH groups (*p* < 0.001) (Figure 7A,B). Treatment with NACA in the Aβ1-41 peptide-infused group (AD models) showed a significant increase in the SYN expression in the mPFC (Aβ vs. Aβ + N, *p* < 0.001, and Aβ vs. N + Aβ + N, *p* < 0.001) (Figure 7A,B). However, the increase did not reach the control level. No significant (*p* > 0.05) difference was found in the expression of the SYN protein among the NC and SH or Aβ + N and N + Aβ + N groups) (Figure 7A,B).

#### 2.4.4. Synaptophysin Immunostaining in the Prefrontal Cortex 

The brain tissues of the rats that were immunostained with an anti-SYN antibody showed positive immunostaining in the mPFC in all groups. The SYN expression in the mPFC of the Aβ group was less in comparison to all other groups, indicating fewer synapses in them (Figure 7C). The SYN expression in the NACA-treated groups (Aβ + N and N + Aβ + N) was higher than in Aβ1-42 peptide-infused group, which indicates improved synapses in the mPFC.

These findings indicate that SYN expression in mPFC of the rat brain was severely affected after the icv infusion of Aβ1-42 peptide (AD model). Both post- and pre- and post-NACA treatment in the Aβ group reversed these deficits by increasing the expression of SYN. However, they failed to normalize the deficits to the control level.

### 2.5. Aβ Protein Expression in the Hippocampus and Medial Prefrontal Cortex

#### 2.5.1. Aβ Protein Level in the Hippocampus

**Figure 8:**
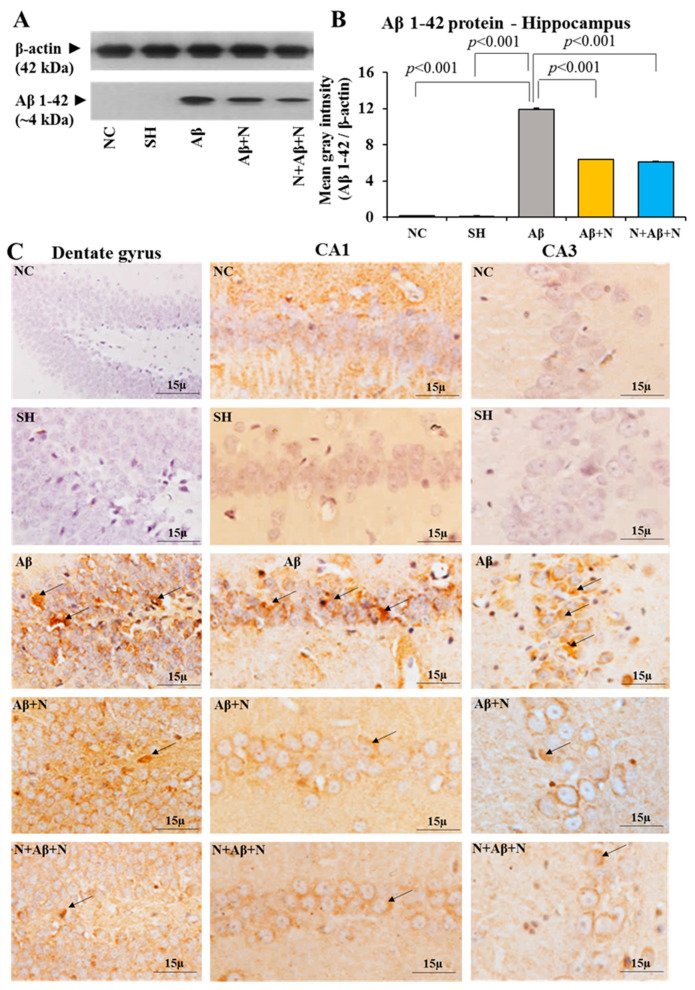
β-amyloid protein content in the hippocampus in the various groups. (**A**) Immunoblot of β-amyloid protein. (**B**) Mean gray intensity (β-amyloid protein/β-actin) of the immunoblots (Mean ± *SEM*). Note the significantly increased Aβ content in the Aβ group in comparison to the NC and SH groups, indicating an increased neurotoxicity. The β-amyloid protein content is significantly decreased in the Aβ + N and N + Aβ + N groups in comparison to the Aβ group (*n* = 6 in all groups). (**C**) Photomicrographs of the dentate gyrus, CA1 and CA3 regions of the hippocampus showing the expression of Aβ protein (arrows) in the various groups. Note the increased expression of Aβ in the Aβ group compared to the NC and SH groups, and the decreased expression in the Aβ + N and N + Aβ + N groups compared to the Aβ group in all regions.

There was a significant elevation of Aβ protein expression in the hippocampal tissue of the Aβ group compared to both the NC and SH groups of rats (Aβ vs. NC/SH, *p* < 0.001) (Figure 8A,B). Treating the Aβ-infused rats (AD models of rats) with NACA, both post, both pre and post Aβ infusion (Aβ + N and N + Aβ + N groups) resulted in a significant reduction of Aβ expression in the hippocampus (Aβ vs. Aβ + N, *p* < 0.001 and Aβ vs. N + Aβ + N, *p* < 0.001) (Figure 8A,B). However, NACA treatment did not decrease the Aβ protein content to the levels seen in the NC or SH groups. No significant (*p* > 0.05) difference was found in the Aβ expression between the Aβ + N and N + Aβ + N groups. The post- or pre- and post-treatment with NACA did not show any difference in Aβ expression in the hippocampus of the rat brain (Figure 8A,B).

#### 2.5.2. Aβ Immunostaining of the Hippocampus 

Rat brain sections immunostained with anti-Aβ antibody showed the highest expression of Aβ in the Aβ1-41 peptide-infused group, and its expression was decreased in the Aβ + N and N + Aβ + N groups in the dentate gyrus crest region, CA1 and CA3 regions (Figure 8C), dentate hilus and supra- and infrapyramidal blade regions (Supplementary Figure S2) in comparison to the Aβ group. The NC and SH groups showed no expression of Aβ.

The Western blot analysis data and immunostaining findings indicate that Aβ expression in the hippocampal region was severely increased after Aβ treatment (AD model). Post- or pre- and post-NACA treatment in the Aβ group reversed these deficits by decreasing the expression of Aβ. However, they failed to normalize these deficits to the level of the control group.

#### 2.5.3. Aβ Protein Level in the Medial Prefrontal Cortex 

**Figure 9:**
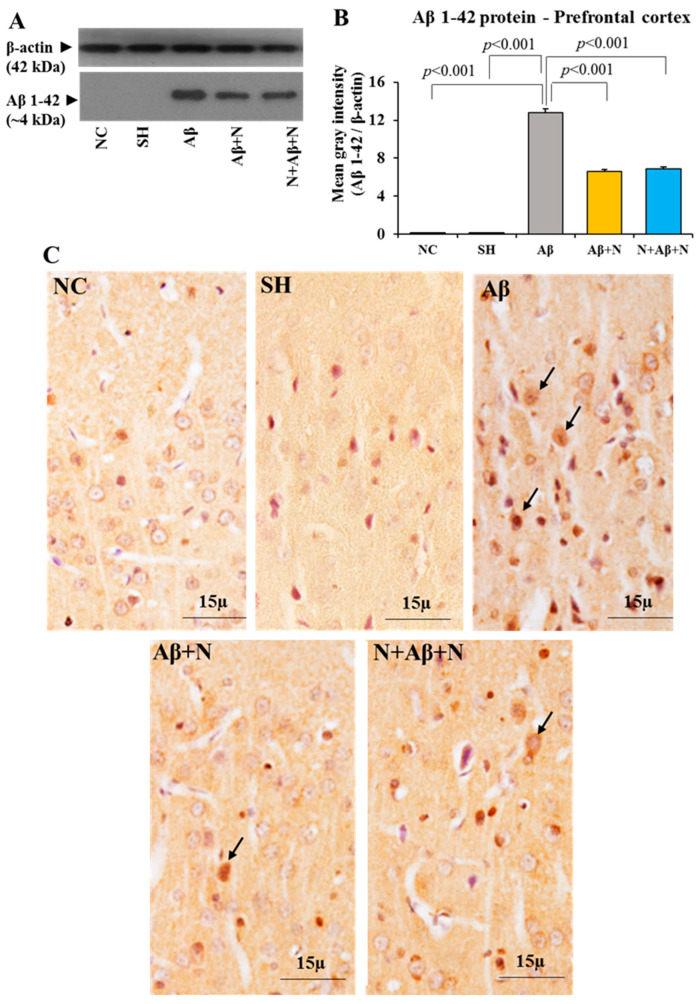
β-amyloid protein content in the medial prefrontal cortex in the various groups. (**A**) Immunoblot of β-amyloid protein. (**B**) Mean gray intensity (β-amyloid protein/β-actin) of the immunoblots (Mean ± *SEM*). Note the significantly increased Aβ content in the Aβ group in comparison to the NC and SH groups, indicating increased neurotoxicity. The β-amyloid protein content is significantly decreased in the Aβ + N and N + Aβ + N groups in comparison to the Aβ group (*n* = 6 in all groups). (**C**) Photomicrographs of medial prefrontal cortex showing the expression of Aβ (arrows) in various groups. Note the increased expression of Aβ in the Aβ group compared to the NC and SH groups, and the decreased expression in the Aβ + N and N + Aβ + N groups compared to the Aβ group.

There was a significant elevation of Aβ protein expression in the mPFC of the Aβ group in comparison to either the NC or SH groups (*p* < 0.001) (Figure 9A,B). Treatment with NACA post and both pre and post icv Aβ infusion (AD models) significantly (*p* < 0.001) diminished the Aβ expression in mPFC when compared to the Aβ group. However, the Aβ content did not reach the baseline levels seen in the NC or SH groups. No significant (*p* > 0.05) difference was found in the expression of Aβ among the Aβ + N and N + Aβ + N groups. The pre- or pre- and post-treatment with NACA did not show any difference in the expression of Aβ in the mPFC region (Figure 9A,B).

#### 2.5.4. Aβ Immunostaining of the Medial Prefrontal Cortex 

Rat brain sections immunostained with an anti-Aβ antibody showed positive immunostaining in the mPFC in all groups. The expression of Aβ in the mPFC in the Aβ group was more compared to all other groups, indicating increased Aβ pathology in them (Figure 9C). The expression of Aβ in the NACA-treated groups (Aβ + N and N + Aβ + N) was lesser than in the Aβ1-42 peptide-infused group which indicates decreased Aβ pathology in the mPFC.

These findings indicate that the Aβ expression in mPFC of the rat brain was severely affected after the icv infusion of Aβ1-42 peptide (AD model). Both pre- and pre- and post-NACA treatment in the Aβ group reversed these deficits by decreasing the expression of Aβ. However, they failed to normalize the deficits to the control level.

### 2.6. Tau immunostaining of the Hippocampus and Medial Prefrontal Cortex

#### 2.6.1. Tau Immunostaining of the Hippocampus

**Figure 10:**
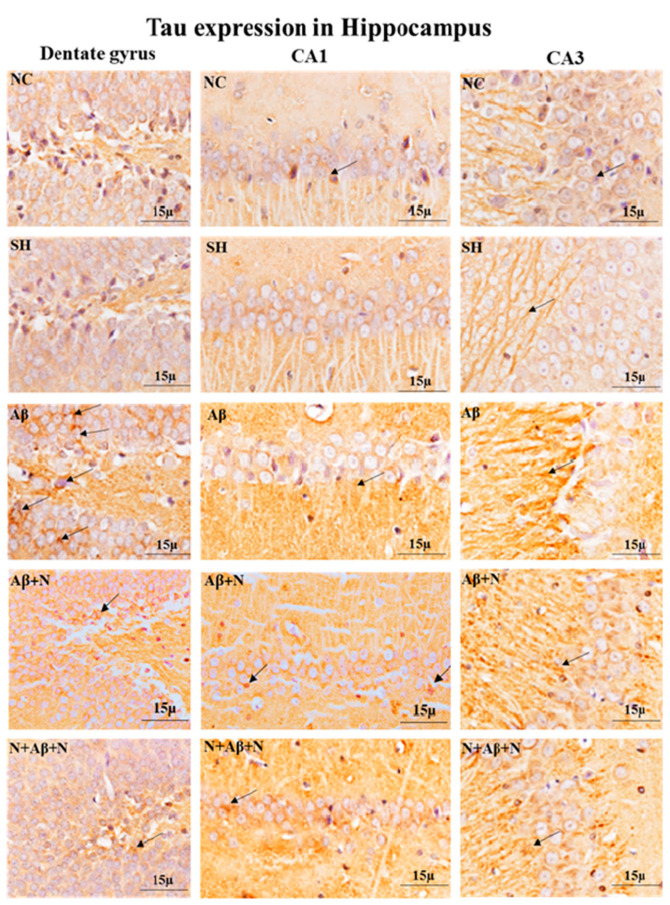
Photomicrographs of dentate gyrus, CA1 and CA3 regions of the hippocampus showing the expression of Tau protein (arrows) in the various groups. Note the increased expression of Tau in the Aβ group compared to the NC and SH groups, and the decreased expression in Aβ + N and N + Aβ + N groups compared to the Aβ group in all regions.

Immunostaining of brain sections with anti-Tau antibody showed increased expression of Tau in the dentate gyrus crest, CA1 and CA3 regions (Figure 10), dentate hilus and supra- and infrapyramidal blade regions (Supplementary Figure S3) in the Aβ group in comparison to the NC/SH groups, indicating a progression of the disease. The Tau-positive immunostaining was decreased in the NACA-treated groups (Aβ + N and N + Aβ + N).

#### 2.6.2. Tau Immunostaining of the Medial Prefrontal Cortex

**Figure 11:**
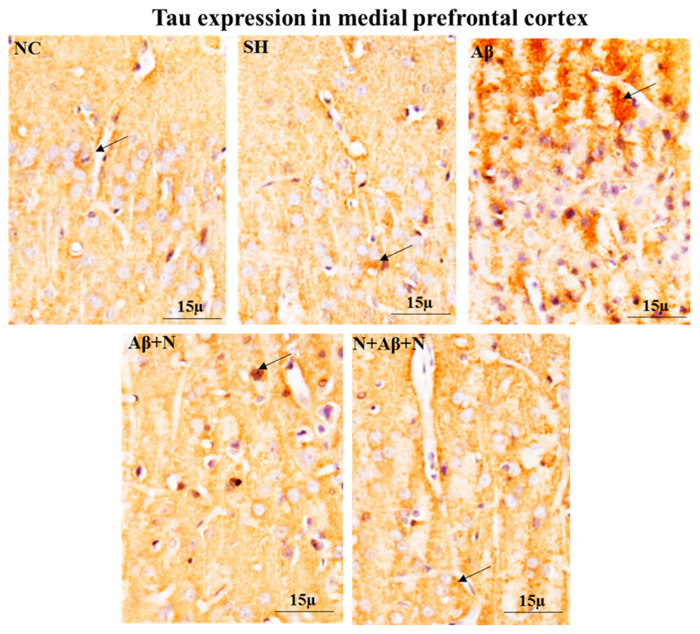
Photomicrographs of medial prefrontal cortex showing the expression of Tau (arrows) in various groups. Note the increased expression of Tau in the Aβ group compared to the NC and SH groups, and the decreased expression in the Aβ + N and N + Aβ + N groups compared to the Aβ group.

Immunostaining of brain tissues using anti-Tau antibody showed increased Tau expression in the mPFC in the Aβ group compared to either the NC or SH groups, indicating Tau pathology by icv Aβ infusion. The Tau-positive immunostaining was decreased in the NACA-treated groups (Aβ + N and N + Aβ + N) (Figure 11).

### 2.7. Cresyl Violet Staining and NeuN Immunostaining 

#### 2.7.1. Cresyl Violet Staining and NeuN Immunostaining of the Hippocampus

**Figure 12:**
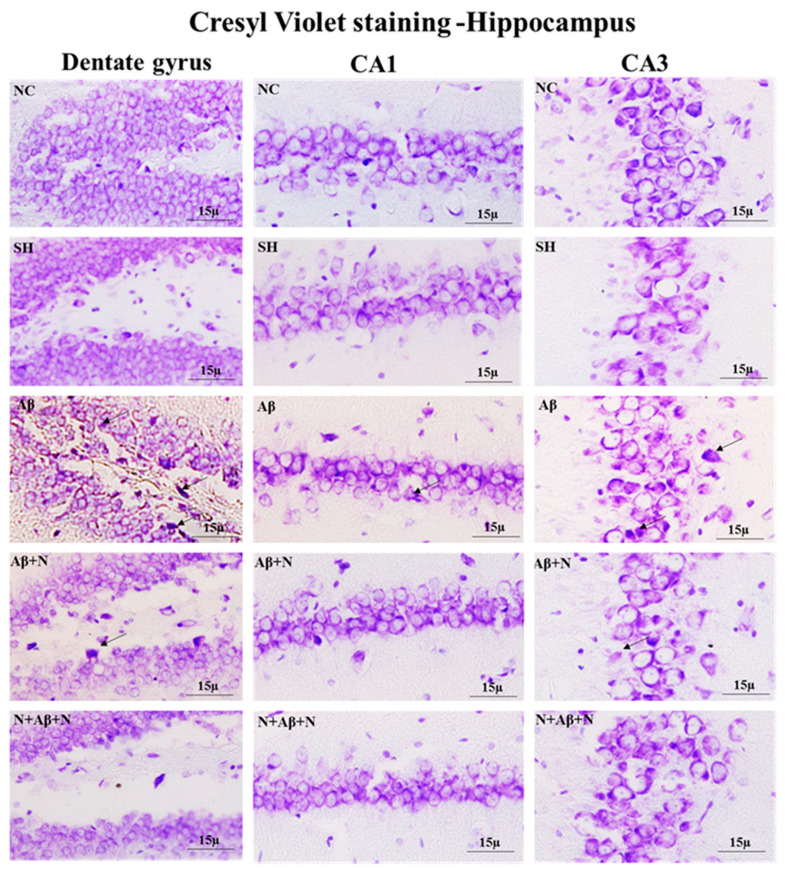
Photomicrographs of dentate gyrus, CA1 and CA3 regions of the hippocampus stained with cresyl violet stain. Note the degenerated neurons (arrow) in the Aβ group in all regions.

Cresyl violet staining showed neuronal death in the dentate gyrus, CA1 and CA3 regions (Figure 12), dentate hilus and supra- and infrapyramidal blade regions (Supplementary Figure S4) in the icv Aβ1-42 peptide-infused group (the Aβ group). NACA treatment pre- and pre- and post-Aβ1-42 peptide infusion reduced neuronal loss.

**Figure 13:**
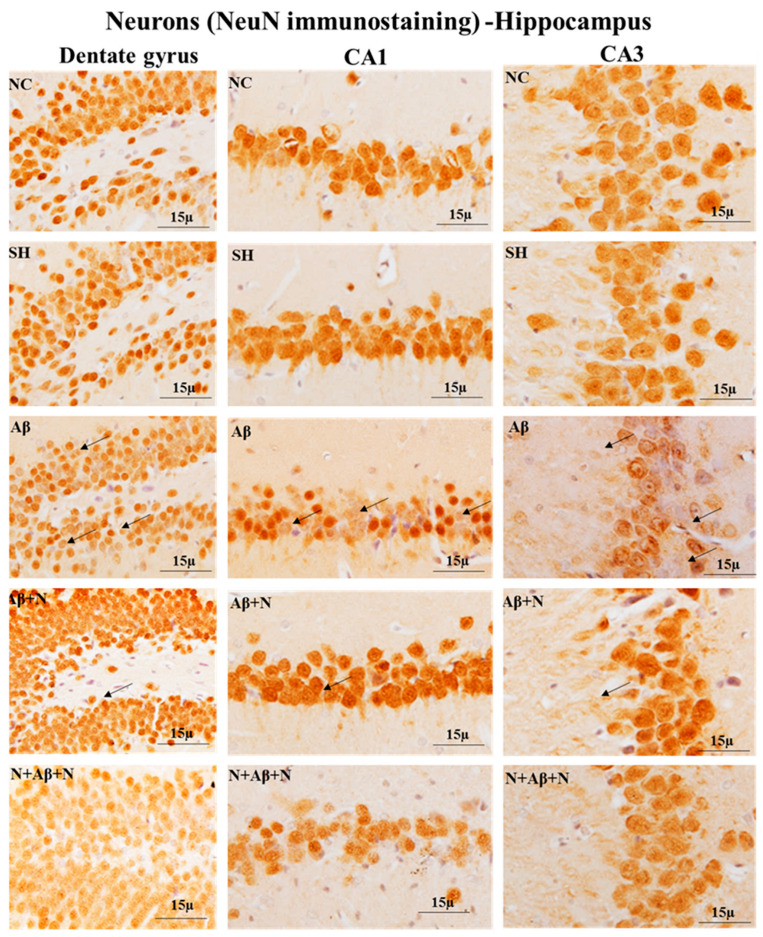
Photomicrographs of dentate gyrus, CA1 and CA3 regions of the hippocampus immunostained with anti-NeuN antibody to demonstrate the neurons. Note the degenerated neurons (arrow) in the Aβ group in all regions.

NeuN immunostaining for neurons showed decreased mature neuronal density in the dentate gyrus, CA1 and CA3 hippocampal regions (Figure 13), dentate hilus and supra- and infrapyramidal blade regions (Supplementary Figure S5) in the icv Aβ1-42 peptide-infused group. NACA treatment pre and pre and post Aβ1-42 peptide infusion protected neurons from degeneration.

#### 2.7.2. Cresyl Violet Staining and NeuN Immunostaining of the Medial Prefrontal Cortex

**Figure 14:**
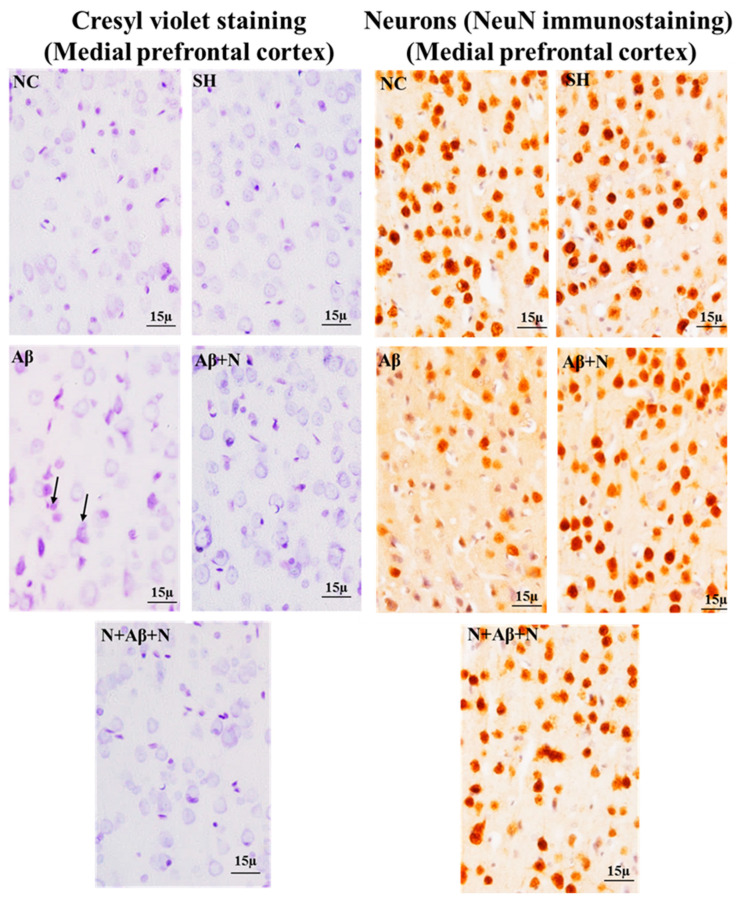
Photomicrographs of medial prefrontal cortex stained with cresyl violet stain (**left** panel) and immunostained with anti-NeuN antibody to demonstrate the neurons (**right** panel). Note the degenerated neurons (arrow) in the Aβ group in all regions.

Cresyl violet staining showed neuronal death, shrunken and deformed neurons in the mPFC in icv Aβ1-42 peptide infused-group (the Aβ group). NACA treatment pre and pre and post Aβ1-42 peptide infusion reduced the neuronal loss in the mPFC (Figure 14).

NeuN immunostaining for neurons showed decreased mature neuronal density in dentate mPFC in icv Aβ1-42 peptide-infused group (Figure 14). NACA treatment pre and pre and post Aβ1-42 peptide infusion protected neurons from degeneration.

### 2.8. GFAP and Iba1Immunostaining of the Hippocampus

#### 2.8.1. GFAP Immunostaining of the Hippocampus

**Figure 15:**
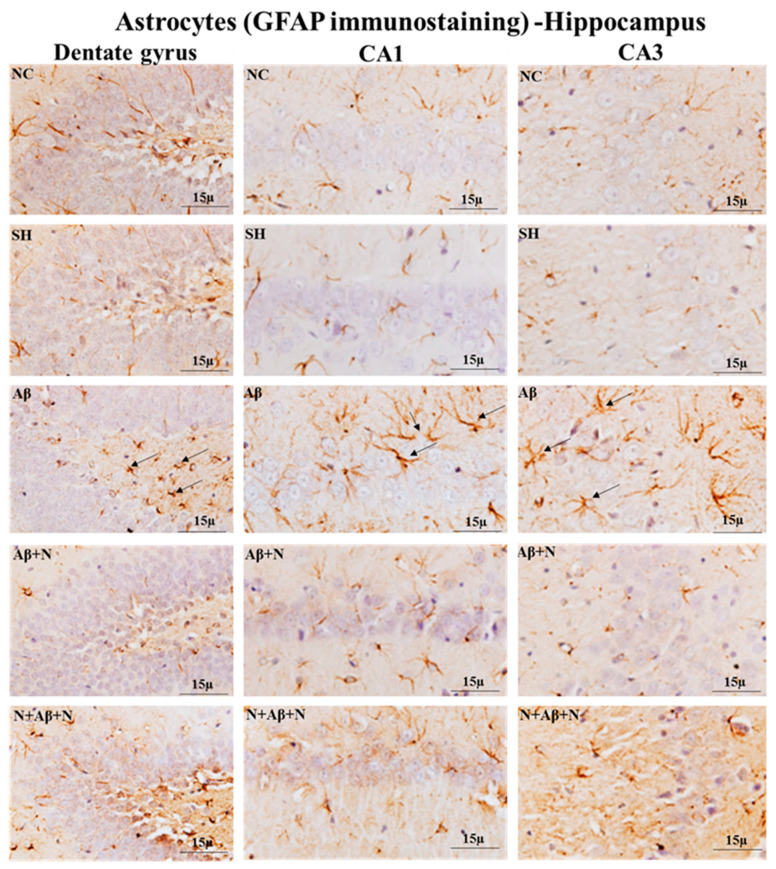
Photomicrographs of dentate gyrus, CA1 and CA3 regions of the hippocampus immunostained with anti-GFAP antibody to demonstrate the astrocytes. Note the elevated number of astrocytes (arrow) in the Aβ group in all regions.

Immunostaining brain sections for astrocytes with an anti-GFAP antibody showed an increased number of GFAP-positive astrocytes in the dentate gyrus, CA1 and CA3 regions (Figure 15), dentate hilus and supra- and infrapyramidal blade regions (Supplementary Figure S6) in the icv Aβ1-42 peptide-infused rats (the Aβ group), indicating astrocytic activation. Treatment with NACA post and pre and post Aβ1-42 peptide infusion (the Aβ + N and N + Aβ + N groups) prevented astrocytic activation in all hippocampal regions.

#### 2.8.2. Iba1 Immunostaining of the Hippocampus

**Figure 16:**
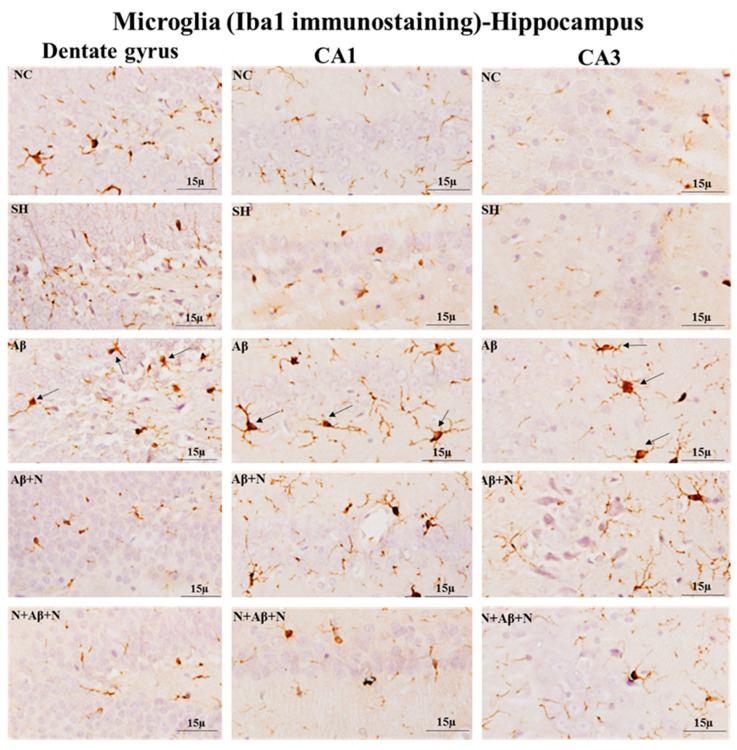
Photomicrographs of the dentate gyrus, CA1 and CA3 regions of the hippocampus immunostained with anti-Iba1 antibody to demonstrate the microglia. Note the elevated number of microglia (arrow) in the Aβ group in all regions.

Immunostaining brain sections for microglia with an anti-Iba1 antibody showed an increased number of Iba1-positive microglia in the dentate gyrus, CA1 and CA3 regions (Figure 16), dentate hilus, suprapyramidal blade and infrapyramidal blade regions (Supplementary Figure S7) in the icv Aβ1-42 peptide-infused rats (the Aβ group), indicating microglial activation. Treatment with NACA post and pre and post Aβ1-42 peptide infusion (the Aβ + N and N + Aβ + N groups) prevented microglial activation in all hippocampal regions.

#### 2.8.3. GFAP Immunostaining of the Medial Prefrontal Cortex

**Figure 17:**
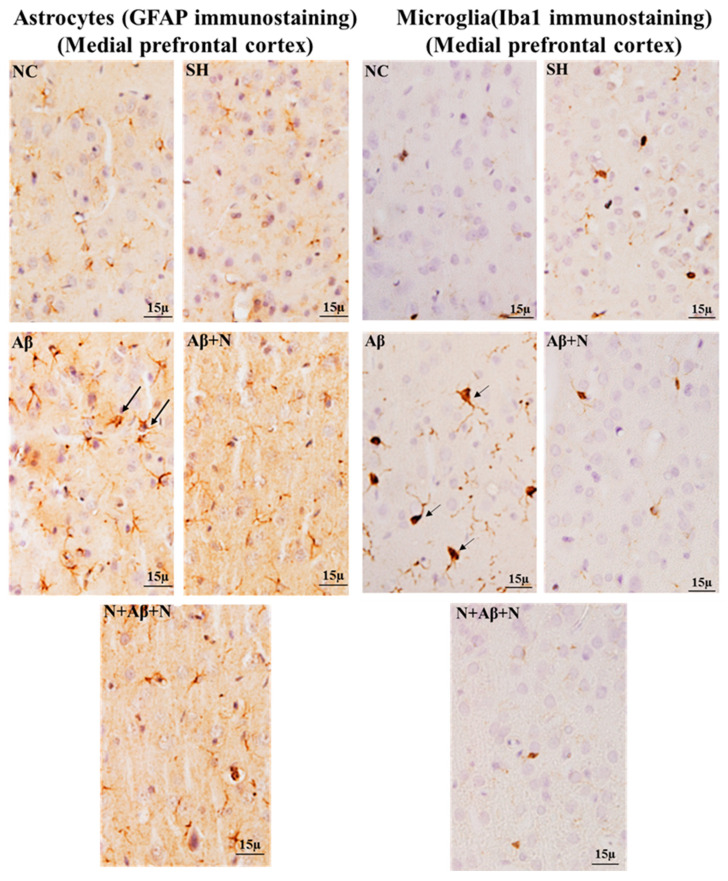
Photomicrographs of medial prefrontal cortex immunostained with anti-GFAP antibody to demonstrate the astrocytes (**left** panel) and immunostained with anti-Iba1 antibody to demonstrate the microglia (**right** panel). Note the elevated number of astrocytes and microglia (arrow) in the Aβ group.

Immunostaining the brain sections for astrocytes with an anti-GFAP antibody showed an increased number of GFAP-positive astrocytes in the mPFC in the icv Aβ1-42 peptide-infused rats (the Aβ group), indicating astrocytic activation. Treatment with NACA post and pre and post Aβ1-42 peptide infusion (the Aβ + N and N + Aβ + N groups) prevented astrocytic activation in the mPFC (Figure 17).

#### 2.8.4. Iba1 Immunostaining of the Medial Prefrontal Cortex

Immunostaining brain sections for microglia with an anti-Iba1 antibody showed an increased number of Iba1-positive microglia in the mPFC of rats in the Aβ group, indicating microglial activation. Treatment with NACA post and pre and post Aβ1-42 peptide infusion (the Aβ + N and N + Aβ + N groups) prevented microglial activation in the mPFC (Figure 17).

### 2.9. Oxidants and Antioxidant Levels

#### 2.9.1. MDA Level in the Hippocampus

**Figure 18:**
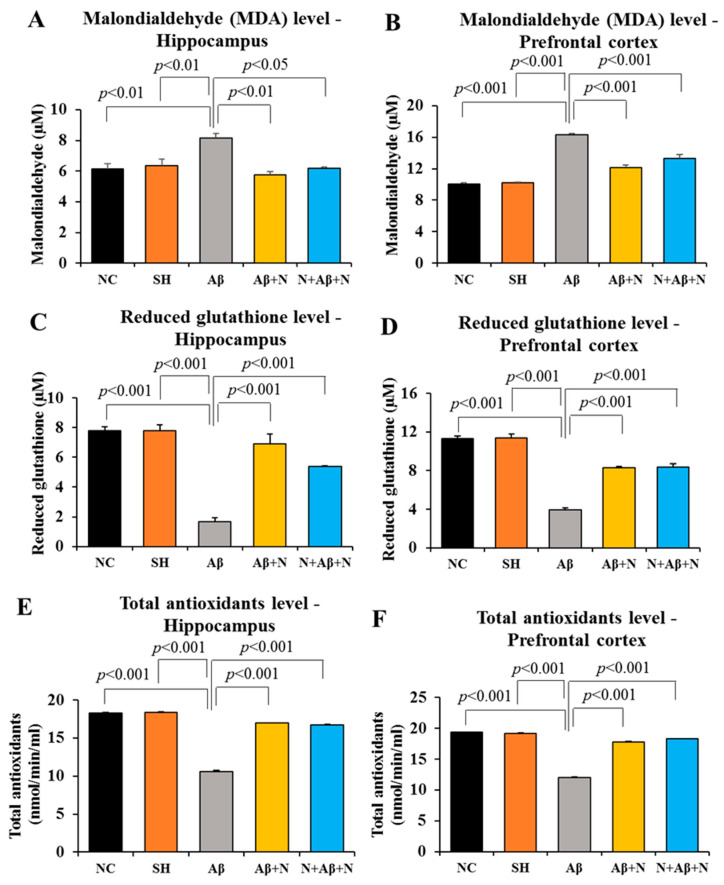
(**A**,**B**) MDA levels (µM) in the hippocampus (**A**) and prefrontal cortex (**B**). Note the significantly increased level of MDA in the Aβ group compared to the NC and SH groups, and the significantly decreased levels in the Aβ + N and N + Aβ + N groups in comparison to the Aβ group in both regions (*n* = 6 in each group). (**C**,**D**) Reduced GSH levels (µM) in the hippocampus (**C**) and medial prefrontal cortex (**D**). Note the significantly decreased level of the reduced GSH in the Aβ group in comparison to the NC and SH groups, and the significantly increased levels in the Aβ + N and N + Aβ + N groups in comparison to the Aβ group in both regions (*n* = 6 in each group). (**E**,**F**) Total antioxidants levels in (nmole/μL) in the hippocampus (**E**) and medial prefrontal cortex (**F**). Note the significantly decreased level of total antioxidants in the Aβ group in comparison to the NC and SH groups, and the significantly elevated levels in the Aβ + N and N + Aβ + N groups in comparison to the Aβ group (*n* = 6 in each group).

MDA, as an end product of lipid peroxidation, is used as a marker for OS. The icv Infusion with Aβ1-42 peptide in the Aβ group (AD model) significantly increased the MDA level in the hippocampus compared to both the NC and SH groups of rats (*p* < 0.01) (Figure 18A). Treatment with NACA, post and pre and post icv Aβ1-42 peptide infusion, has resulted in a significant reduction in the MDA level in the hippocampus compared to the Aβ group (Aβ vs. Aβ + N, *p* < 0.01 and Aβ vs. N + Aβ + N, *p* < 0.05) (Figure 18A). No significant (*p* > 0.05) difference was found in the levels of MDA in the hippocampus between the Aβ + N and N + Aβ + N groups.

#### 2.9.2. MDA Level in The medial Prefrontal Cortex

There was a significant elevation in the MDA level in the mPFC of the Aβ group compared to both the NC and SH groups (*p* < 0.001) (Figure 18B). Treatment with NACA, both post and pre and post Aβ1-42 peptide infusion, significantly reduced the MDA level in the mPFC compared to the Aβ group (Aβ vs. Aβ + N, *p* < 0.001 and Aβ vs. N + Aβ + N, *p* < 0.001) (Figure 18B). However, treatment with NACA did not reduce the MDA level to the baseline levels seen in the NC or SH groups (NC vs. Aβ + N, *p* < 0.05 and SH vs. N + Aβ + N, *p* < 0.05). No significant (*p* > 0.05) difference was found in MDA expression between the Aβ + N and N + Aβ + N groups (Figure 18B).

#### 2.9.3. Reduced GSH Level in the Hippocampus

There was a significant reduction in the educed GSH level in hippocampus of the Aβ group in comparison to the NC/SH groups of rats. Treatment with NACA, post and pre and post ivc infusion of Aβ1-42 peptide, significantly elevated the reduced GSH level in the hippocampus compared to the Aβ group (Aβ vs. Aβ + N, *p* < 0.001 and Aβ vs. N + Aβ + N, *p* < 0.001) (Figure 18A,C). No significant (*p* > 0.05) difference was found in the reduction of GSH expression between the Aβ + N and N + Aβ + N groups (Figure 18C).

#### 2.9.4. Reduced GSH Level in the Medial Prefrontal Cortex

There was a significant reduction in the level of reduced GSH in the mPFC of the Aβ group in comparison to the NC/SH groups of rats. Treatment with NACA, post and pre and post ivc Aβ1-42 peptide infusion, significantly elevated the reduced GSH level in the mPFC compared to the Aβ group (Aβ vs. Aβ + N, *p* < 0.001 and Aβ vs. N + Aβ + N, *p* < 0.001). However, the treatment with NACA did not increase the reduced GSH to the levels seen in the NC or SH groups (NC vs. Aβ + N, *p* < 0.001 and NC vs. N + Aβ + N, *p* < 0.001). No significant (*p* > 0.05) difference was found in the levels of reduced GSH between the Aβ + N and N + Aβ + N groups (Figure 18D).

#### 2.9.5. Total Antioxidants Level in the Hippocampus

There was a significant reduction in the level of total antioxidants in the hippocampus of the Aβ group in comparison to the NC/SH groups of rats (*p* < 0.01). Treatment with NACA, post and pre and post icv Aβ1-42 peptide infusion, significantly elevated the total antioxidants level in the hippocampus compared to the Aβ group (Aβ vs. Aβ + N, *p* < 0.05 and Aβ vs. N + Aβ + N, *p* < 0.05). No significant (*p* > 0.05) difference was found in total antioxidants level between the Aβ + N and N + Aβ + N groups (Figure 18E).

#### 2.9.6. Total Antioxidants Level in the Medial Prefrontal Cortex

There was a significant reduction in level of total antioxidants in the mPFC of the Aβ group in comparison to the NC/SH groups of rats (*p* < 0.001) (Figure 18F). Treatment with NACA, post and pre and post icv Aβ1-42 infusion significantly (*p* < 0.001) elevated the total antioxidant level in mPFC compared to the Aβ group (Aβ vs. Aβ + N, *p* < 0.001, Aβ vs. N + Aβ + N, *p* < 0.001) (Figure 18F). However, this elevation did not reach the baseline levels seen in the NC or SH groups (NC vs. Aβ + N, *p* < 0.001, NC vs. N + Aβ + N, *p* < 0.001). No significant (*p* < 0.05) elevation was seen in the level of total antioxidants in the N + Aβ + N group in comparison to the Aβ + N group (Aβ + N vs. N + Aβ + N, *p* < 0.001) (Figure 18F).

## 3. Discussion

This study evaluated the potential protective effects of NACA in an animal model of AD. We observed that intraventricular Aβ1-42 peptide administration induced AD-like changes, including significant cognitive impairment as well as substantial neuronal loss, tau pathology, β-amyloid pathology, downregulation of synaptogenesis and gliosis in the prefrontal cortex and hippocampus of rats. NACA treatment effectively attenuated these abnormalities and normalized the condition towards the sham or control group. The administration of NACA prior to the AD pathology seems to work proficiently in a few of the parameters studied. These findings provide a mechanistic basis for the protective effects of NACA on AD-type neurodegeneration.

### 3.1. NACA Reversed Oxidative Stress-Induced Cognitive Dysfunction

The passive avoidance and Morris water maze tests are well-established behavior study models for evaluating cognitive functions in animals. In the present study, both these study models demonstrated cognitive dysfunction after the icv administration of Aβ1-42 peptide. In the Morris water maze test, animals that received Aβ1-42 peptide took a longer time to reach the platform and also travelled further before reaching the platform during the evaluation of learning. During the evaluation of the memory, these animals spent less time in the platform quadrant, traveled less distance in the platform quadrant and had increased latency to reach the platform quadrant. In a passive avoidance test, the rats received a foot shock in the dark compartment during the last trial/learning session. When a memory retention test is conducted 24 h after a foot shock, the normal rats tend to avoid entering the dark compartment and tend to spend more time in the bright compartment using their previous memory of the foot shock they received. The rats that received Aβ1-42 peptide spent longer in the dark compartment despite having received foot shocks previously, indicating a severe memory impairment. Thus, from these two study models, it is clear that the intracerebroventricular administration of Aβ1-42 peptide causes both learning and memory deficits. Our primary findings from the behavioral studies are consistent with previous studies where an icv Aβ injection resulted in cognitive dysfunction [33,34]. NACA treatment, either prophylactic (before and after inducing AD pathology) or restorative (only after inducing AD pathology), reversed the cognitive deficits in the form of learning and memory in the Morris water maze as they took less time and travelled less distance to reach the platform during learning sessions. They also had reduced latency to reach the platform quadrant and spent more time and traveled more distance in the platform quadrant during the memory retention test. In the passive avoidance test, both the prophylactic and restorative administration of NACA reversed the learning and memory deficits as they spent more time in the bright compartment during the memory retention test to avoid foot shocks in the dark compartment. Recently, Kim et al. [35] found that NACA exerted its antioxidant potential against kainic acid-induced OS in aging organotypic hippocampal slice cultures. Apart from this research, we found no other study that addressed the antioxidant effect of NACA against Aβ-induced neurotoxicity. The present study is the first of this kind where NACA has reversed AD-like pathology in rats by upregulating the antioxidant defense.

The cognitive dysfunction was associated with severe OS. Aβ1-42 peptide administered rats showed a high level of MDA in both the hippocampus and the mPFC. Further, these rats showed a decreased level of reduced GSH and total antioxidants in both the hippocampus and mPFC. These results established a direct link between the cognitive deficits observed in this study with OS. Similar results were reported in aged rats where cognitive dysfunction was associated with OS [36]. OS in brain tissue is well known to cause neurodegenerative disorders and it has a central role in the direct initiation of neurodegeneration.

Lipid peroxidation is often assayed by measuring TBARS. The end products of lipid peroxidation, such as MDA level estimation, were broadly used to indicate OS in many studies [37]. Lipid peroxidation is the process by which free radicals “steal” electrons from lipids in the cellular membrane leading to the destruction of the cell. This process is succeeded by a free radical chain reaction. It always affects polyunsaturated fatty acids in the brain. Further, we know that the end products of lipid peroxidation are dangerous and can be mutagenic and carcinogenic. For example, the MDA reacts with deoxyadenosine and deoxyguanosine in DNA, forming DNA adducts with them. Oxidative damage to lipids and proteins is an initial event in inducing neurodegenerative diseases. In the present study, both the prophylactic and restorative treatment of NACA was able to downregulate MDA levels in both the hippocampus and mPFC. The antioxidant benefit of NACA was better pronounced in the hippocampal region. This could be due to the different mechanisms of NACA on the hippocampal and prefrontal tissues.

GSH contains glutamic acid, cysteine and glycine. Its mechanism of action is to decrease inactive disulfide linkages of enzymes to the active sulfhydryl group while the sulfhydryl group of GSH becomes oxidized. In this way, GSH exerts a vital function in the defense against membrane peroxidation by decreasing hydrogen peroxide with GSH peroxidase. In cells, GSH is retained in its reduced form by GSH reductase. It decreases other metabolites and enzymes as well as reacting directly with oxidants. Reduced GSH is necessary for the cellular detoxification of ROS in brain cells [38]. In the present study, both the prophylactic and restorative treatment of NACA was able to upregulate reduced GSH levels in both the hippocampal and the mPFC tissues.

The major advantage of evaluating the total antioxidants is to evaluate the antioxidant potential of all antioxidants in each tissue and not just the antioxidant capacity of a single component [39]. In our study, both prophylactic and restorative NACA treatment was able to increase the total antioxidant level in both the hippocampal and mPFC tissues. In the mPFC, the prophylactic therapy had better effects compared to restorative therapy. This OS-induced neuronal dysfunction involves several well-known mechanisms which include Aβ pathology, Tau hyperphosphorylation with NFTs formation within the neurons, loss of synaptic connections, reduced neurogenesis and eventually neuronal loss. These pathologies are directly linked with cognitive dysfunctions. All these pathologies associated with AD are further explored in the present study.

### 3.2. NACA Downregulated Oxidative Stress-Induced Aβ Pathology

It is not certain whether the accumulation of β-amyloid is a consequence or an actual cause of OS. Both OS and amyloid pathology are linked to each other since Aβ induces OS, and OS increases Aβ deposition. Aβ plaques are responsible for impaired synaptic plasticity, neuroinflammation, cholinergic denervation, dendritic alterations, synaptic loss and substantial neuronal loss via OS [40]. Most researchers believe that a prolonged period of steady oxidative damage precedes and results in the appearance of clinical and pathological symptoms of AD, including the deposition of Aβ, formation of NFT, metabolic dysfunction and cognitive deficit [41]. Additionally, many antioxidants were found to prevent the formation and extension of Aβ fibrils in vitro. During the Aβ plaque formation, the Aβ protein generates hydrogen peroxide. The Aβ protein induces OS in many forms (lipid peroxidation, DNA oxidation, glycoxidation), and this is responsible for the degeneration of synapses [42]. Many transgenic animal models simulating AD-like Aβ abnormalities have been effectively established, where cognitive dysfunctions associated with the accumulation of Aβ proteins inside and outside neurons were confirmed. Leon and his co-workers demonstrated intraneuronal Aβ protein accumulation in transgenic mice, which was extensive in the hippocampus and most cortical areas [43]. In the present study, Aβ1-42 peptide-treated animals had increased Aβ protein levels in both their hippocampus and mPFC. Further, the IHC study showed a higher number of expressions of Aβ-positive neurons in all hippocampal regions and also in the mPFC. We also found both a diffuse variety of plaques as well as dense-cored plaques in some. This is not surprising as we have directly administered Aβ1-42 peptide to the lateral ventricle, which has resulted in increased expression of this peptide and may also be due to a lack of its clearance. The plaques and Aβ-positive neurons were present on the other half of the brain where Aβ1-42 peptide were not injected. Both prophylactic and restorative NACA treatment resulted in a reduced number of Aβ proteins in both the hippocampus and mPFC. Further, the IHC study showed similar changes in all regions of the hippocampus as well as the mPFC. This demonstrates the ability of NACA to reduce the number of abnormal beta-amyloid proteins either by suppressing the misfolding of this protein or by enhancing its clearance.

### 3.3. NACA Reversed Oxidative Stress-Induced Tau and NFT Expression

In AD, the increased phosphorylation of particular amino acids in Tau triggers the dissociation of proteins from microtubules, unsettling the transport structure and causing neuronal starvation and death. Thus, Tau hyperphosphorylation has a crucial role in NFT changes within neurons, as well as AD pathogenesis [44,45]. The OS has been shown to promote the hyperphosphorylation of Tau [3,46]. Currently, OS is considered as a further hallmark of Tau pathology in animal models and AD patients. Aβ accumulation is responsible for AD-related pathology, including the initiation of NFTs, and eventually neuronal death [47]. The Aβ1-42 peptide administration has accelerated Tau pathology in vitro and in vivo when compared to Aβ1-40 [48]. It is still unclear whether OS is an early causative factor, or a consequence of the cellular damage caused by the hyperphosphorylation of the Tau protein. In the present study, the icv administration of Aβ1-42 peptide caused an excess expression of hyperphosphorylated Tau in the hippocampus and the mPFC in Western blotting analysis. The IHC study also revealed many NFTs or hyperphosphorylated Tau-positive neurons in all hippocampal areas and the mPFC. We conclude that the increased expression of Tau in the neurons of the hippocampus and mPFC is correlated with cognitive impairment. These intraneuronal phosphorylated Tau proteins inhibit axonal transportation, neural transmission and synaptic activity, resulting in cognitive dysfunction. Hyperphosphorylated Tau in neurons is strongly correlated with AD patients [49]. In AD, Tau pathology builds up in a sequential manner from the entorhinal cortex to the hippocampus, frontal and temporal cortices and finally to all isocortices. It is still unknown why there are different susceptibilities in particular brain areas to Tau pathology. It might be caused by the region-specific expression of Tau pathology-related proteins, such as Tau phosphatases and kinases. Lace et al. [50] showed Tau pathology specifically in hippocampal input areas and projection zones such as the entorhinal cortex, CA1 region and the molecular layer of the dentate gyrus. A dense expression of Tau was found at the junction between CA1 and subiculum. In turn, tauopathy is believed to progressively spread to the hippocampus. Accordingly, the perforant pathway is the main target region, which includes the dentate gyrus, CA3, CA1 and subiculum [51]. Another study showed remarkable Tau hyperphosphorylation in the typical thorny excrescences of the neurons of the CA3 region [52]. Since thorny outgrowths represent a major synaptic target of granule cell axons (mossy fibers), abnormal phosphorylation likely plays a role in the memory impairment seen in patients with AD. In this study, both prophylactic and restorative NACA treatment minimized the expression of hyperphosphorylated Tau in terms of quantitative analysis (western blot) and qualitative analysis (IHC). From these data, it is very evident that antioxidant defense within the brain will certainly provide a defense against Tau pathology-mediated cognitive dysfunction. Few studies have demonstrated the antioxidant effect on the downregulation of NFTs. The administration of vitamin E to the rats significantly reversed the scopolamine-induced density increase of the Aβ plaques and NFTs in the hippocampus [53].

### 3.4. NACA Ameliorated Oxidative Stress-Induced Downregulation of Synaptophysin

The formation of synapses occurs by inter-neuronal connections, which allow for the passing of an electrical or chemical signal from one neuron to another. Most neurodegenerative diseases cause damage or loss to this passage. It is generally understood that impairment and loss of synapses contribute to cognitive declines in AD patients. Synaptic transmission critically depends on numerous cellular mechanisms, including biosynthesis and the delivery of neurotransmitters to locations of synapses that require intact tracts of microtubules. Synaptic loss in the affected areas of the brain correlates best with cognitive dysfunction in patients with AD and has been considered the early mechanism that precedes the loss of neurons [54]. Many in vivo and in vitro research has established a direct relationship between OS and synaptic dysfunction in AD [55]. The loss of the presynaptic vesicle protein SYN in the hippocampus correlates with cognitive dysfunction in AD [56,57]. Adams et al. [58] demonstrated that Aβ-42 binds to SYN with picomolar affinity, resulting in the inhibition of both neurotransmitter release and synaptic plasticity. In the present study, rats treated with Aβ1-42 peptide had a lower SYN protein level in both the hippocampus and mPFC. Both prophylactic and restorative treatments of NACA have resulted in increased SYN protein levels in the hippocampus. From our results, it can be established that the enhancement of learning and memory activities is associated with the upregulation of synaptophysin in the hippocampus. A correlation is established between behavior and the intensity of SYN in the hippocampus that receives inputs from the cerebral cortex [59]. The SYN is a reliable indicator of synaptic plasticity, and it correlates well with cognitive dysfunction in AD patients and mouse models with neurodegeneration. The findings indicate that SYN expression was elevated in NACA-treated animals. Another interesting finding of this study is that the level of SYN did not increase or normalize in the mPFC with restorative NACA treatment. This demonstrates that boosting antioxidant defense by means of prophylactic therapy is necessary to have the maximum neuroprotective effects by normalizing the SYN level in the mPFC.

### 3.5. NACA Reversed Oxidative Stress-Induced Reduced Neurogenesis

Adult neurogenesis in subgranular zones of the dentate gyrus and their functional integration into hippocampal neuronal networks are crucial for maintaining functional plasticity. This process of neurogenesis involves a series of processes which include proliferation, differentiation, migration and maturation. This entire process is impeccably sensitive to OS, causing reduced neurogenesis [60,61]. The icv administration of Aβ1-42 peptide was previously shown to have adverse effects on adult neurogenesis [62]. Though our study did not evaluate the effect on the differentiation and migration of newly formed neurons, we observed a deteriorating effect on neuronal proliferation after Aβ1-42 peptide administration. Rats who received Aβ1-42 peptide showed reduced levels of DCX protein in the hippocampus and DCX-positive neurons in the dentate gyrus, indicating reduced neuronal proliferation. Both prophylactic and restorative treatments of NACA elevated DCX protein levels in the hippocampus and DCX-positive neurons in the dentate gyrus, indicating enhanced neuronal proliferation. The magnitude of neuronal proliferation was higher in rats who received prophylactic NACA treatment. As stated previously, though we have not evaluated the migration and functional recruitment of newly formed neurons after NACA treatment, NeuN staining showed enhanced expression of NeuN-positive neurons. NeuN is a marker for matured neurons and the increased expression of these neurons in the CA region of the hippocampus, possibly suggesting the incorporation of newly formed neurons in the functional circuit. From these results, it can be postulated that Aβ1-42 peptide-induced OS causes reduced neuronal proliferation in the dentate gyrus, and this could be one of the contributing factors for cognitive dysfunction. This OS effect on the dentate gyrus’s neuronal proliferation is minimized by NACA treatment. Further study of the effect of NACA on migration and its functional establishment could be addressed in the future. There are few studies demonstrating the antioxidant treatment-induced enhancement of neurogenesis. Anticonvulsant drug topiramate enhances neurogenesis through its antioxidant effect in d-Galactose-induced aging in mice [63]. Another study demonstrated that the antioxidant resveratrol enhanced neurogenesis in lipopolysaccharide-induced depressive behavior in mice [64]. It is generally believed that hippocampal neurogenesis is essential for maintaining learning and memory, depending on the proper function of the hippocampal circuitry. Newly formed neurons in subgranular zones of the dentate gyrus become incorporated into a functional local network, which attributes cognitive functions such as spatial learning.

### 3.6. NACA Reversed Oxidative Stress-Induced Gliosis

The glial cell interaction is important for neurotransmission and this interruption in glial functions also contributes to cognitive impairment. In brain regions where oxygen free radicals and Aβ plaques are present, both microglia and astrocytes become activated [65,66]. It is also observed that microglia and astrocytes are increased in areas near the Aβ plaques in AD [67]. The reactive astrocytes around the Aβ plaques induce inflammatory reactions and alter Ca^2+^ signaling [68,69]. A loss of astrocytic function and reactivity contribute to AD [70]. Our study showed activated astrocytes and an abundant expression of reactive microglia (microgliosis) in almost all areas of the hippocampus and mPFC in the presence of Aβ-positive neurons and elevated Aβ protein levels. The gliosis found in this study is obviously due to Aβ1-42 peptide-induced neuroinflammation and neuronal damage or OS.

The astrocytes are concerned with keeping or processing OS in AD. Thus, the reactive astrocytes seen in our study have caused OS, which was shown by the antioxidant studies. The astrocytosis inhibited axonal transmission, which resulted in cognitive impairment. Also, the overexpression of Aβ proteins seen in our study caused OS in the hippocampus and the prefrontal cortex. It is known that an increased number of Aβ proteins induces OS in the brain [71]. In neurons, astrocytes afford the resources required for GSH production [72]. Though the study did not estimate the Aβ protein expression in astrocytes in particular, overexpression of this protein might have prevented the production of GSH.

The microglia express Aβ plaque degrading enzymes that allow it to phagocytose Aβ plaques. In addition, when they become activated, they produce proinflammatory chemokines, cytokines and neurotoxins [73]. Microglia are suggested to have a dual role in AD pathogenesis [74]. They can eliminate soluble fibrillar Aβ, but their constant interactions with Aβ can cause a neurotoxic inflammatory response. In our study, we demonstrated that the activated microglia in the hippocampus and mPFC regions were minimized with the treatment of NACA in AD rat models. The diminished expressions of these glial cells cannot be positively correlated with the neuroprotection of NACA since gliosis could be the response for Aβ1-42 peptide-induced neuroinflammation. Possibly the NACA might have reduced that neuroinflammation. Since the study parameter did not involve a quantification of these immunostaining or inflammatory markers, we could not figure out the potential differences in NACA treatments (prophylactic vs. restorative). Evaluating the specific markers of neuroinflammation would give a better indication of this. Joy et al. [75] demonstrated the antioxidant efficacy of NAC by inhibiting the activation of microglia in the hippocampal and mPFC tissues of the rat brain in the presence of Aβ-positive neurons [12]. By a similar mechanism, the NACA exerted its neuroprotective efficacy, as suggested by the results of our antioxidant studies. It would be interesting to see how NACA triggers microglia in clearing β-amyloid peptides.

### 3.7. NACA Ameliorated Oxidative Stress-Induced Neuronal Loss

As a result of to its high oxygen consumption, the antioxidant capacity of the brain is reduced. When ROS accumulate, it leads to degeneration of neurons because of a large volume of unsaturated fatty acids in neuronal cell membranes. OS is neurotoxic, as demonstrated by many studies. Moreover, OS is known to be the main factor in causing the neuronal changes and behavioral deficits seen in AD [76,77]. The accumulation of Aβ proteins in neurons and Aβ plaques causes a loss of neurons [78,79]. In our study, we observed severe neuronal loss in almost all hippocampal regions and the mPFC, as seen by CV and NeuN staining. As a result, the cognitive deficits related to memory loss that were observed in our study can be correlated positively. It is a well-established fact that a loss of hippocampal neurons is accompanied by cognitive decline [80,81,82]. Both memory and decision making are processed in the mPFC, but its advanced functions (i.e., learning and memory consolidation) require a good connection with the hippocampus [83]. Also, the mPFC is important for both recent and remote memory [84]. Because the hippocampus plays an important role in memory, it is expected that the hippocampus and mPFC are anatomically related to each other. Both are required to consolidate memory after learning. Moreover, the mPFC is involved in retrieving memories after performing a task for several days [85]. In our study, the foot shock escaping task was done for five days to observe the rat’s ability for memory retrieval. In our study, neuronal loss in the mPFC, along with impaired memory retrieval, was seen in rats after administering Aβ1-42 peptide; however, this effect was reversed by NACA.

Reduced levels of GSH are well-known hallmarks of AD. Several mechanisms were explained for reduced GSH level-induced AD pathologies. Reduced GSH initiates lipid-epoxidizing enzyme 12-lipooxygenase activation, causing apoptosis and the death of neurons. NAC, the GSH precursor, has provided beneficial effects in combating AD pathology through high cytoplasmic cytochrome c release, reduced mitochondrial membrane potential, and reduced Bcl-2 expression, p53 expression and caspase pathways. NACA, a revised form of NAC with a higher membrane and blood–brain barrier permeability, has reduced the degeneration of neurons and apoptosis and increased manganese superoxide dismutase (antioxidant enzyme) levels [85]. NACA provides neuroprotection via the activation of the Nrf2-ARE signaling pathway after traumatic brain injury in mice [86]. A variety of antioxidants (metabolic and mitochondria-targeted) were effective in animal models of AD and a few clinical studies. Our study showed the positive effect of boosting the GSH system in AD-like conditions, but the efficacy of GSH in clinical studies remains inconclusive. In general, antioxidant supplementation is effective in animal studies but not in human trials. However, the results of clinical trials that use NAC as an antioxidant to prevent and treat AD are promising. To summarize, the treatment of NACA has exerted its neuroprotective effect, as seen in several parameters. It has also been observed that the pretreatment of NACA has a better neuroprotective effect in several parameters. Hence, building antioxidant defense during normal aging is likely to delay the signs and symptoms of AD. The parental form of NACA has already been in several clinical trials as a therapeutic strategy. More research concerning the neuroprotective efficacy of NACA could make it an ideal candidate for future clinical trials and therapeutic options. The icv administration of the Aβ1-42 peptide has demonstrated neurotoxicity in rats. This neurotoxicity was observed in the form of cognitive impairment, reduced neurogenesis, reduced SYN, a buildup of Aβ and Tau and a considerable loss of neurons in the hippocampal and mPFC areas of the brain. Further, the neurotoxicity induced by Aβ1-42 peptide was associated with severe OS and gliosis in the hippocampus and mPFC areas of the brain. These cognitive deficits were associated with the upregulation of Aβ-positive-neurons, hyperphosphorylated Tau-positive neurons and NFTs in the hippocampal and mPFC areas, the regions responsible for cognition. The animal model used in the study shows several characteristics of AD pathology. Both prophylactic and restorative treatment methods have shown promising results. The limitation of this study is that only male rats were included to avoid hormonal effects on the results of the study parameters. Sex differences may exist which need to be investigated in future. Another arguable limitation is that the effect of NACA alone in normal rats was not explored, though if one of the experimental groups had NACA treatment for 7 days before inducing AD pathology it would have been interesting to observe its effect. The quantification of tau- and amyloid-positive neurons and their relative density with respect to gliosis shall be considered in future studies.

## 4. Materials and Methods

### 4.1. Animals and Ethics Approval

Two-and-a-half-month-old male *Wistar* rats, bred and maintained at the Animal Resources Center at the Health Sciences Center, Kuwait University were used in the present study. The study was conducted after obtaining animal ethical approval from the Kuwait University Health Sciences Center’s Animal Ethics Committee (Ref:23/VDR/EC/3560 & 3561, dated 7 November 2019). Animals were housed in sterile polypropylene cages in a controlled environment (22 ± 2 °C) with a 12-h light/dark cycle. They were fed standard food and water ad libitum. Animal treatment and maintenance were carried out according to the guidelines of the institutional Animal Care and Use Committee of Kuwait University, which follows the guidelines of the National Institute of Health’s Guide for the Care and Use of Laboratory animals. All efforts were made to minimize the number of animals and their suffering.

### 4.2. Animal Groups and Experimental Design

Rats were randomly divided into five groups and treated as described below (*n* = 18 in each group). (Supplementary Figure S8).

i. **Normal control (NC) group:** The rats in the normal control group were undisturbed in their home cages except for daily intraperitoneal (ip) injection of normal saline (0.5 mL) during the experimental period (seven consecutive days).
ii. **Sham (SH) group:** The rats in the sham group underwent sham stereotaxic surgery as described below and 5 µL of sterile saline was infused into each lateral ventricle (bilateral icv (intracerebroventricular) infusion) via a 10 µL Hamilton micro-syringe. Throughout the study period, these rats remained in their home cages and received daily intraperitoneal injections of normal saline (0.5 mL), as with those rats in the NC group.
iii. **Amyloid-β (Aβ) group:** The rats in this group underwent stereotaxic surgery as described below and 5 µL of sterile Aβ1-42 peptide solution (5 µg Aβ1-42/5 µL sterile saline) was infused into each lateral ventricle. The rats in this group remained in their home cages and received daily ip injections of normal saline (0.5 mL) for seven consecutive days.
iv. **Amyloid-β + N-Acetyl Cysteine Amide (Aβ + N) group:** The rats in this group were infused with 5 µL of Aβ1-42 peptide solution (5 µg Aβ1-42/5µL sterile saline) into each lateral ventricle as in the Aβ group above. However, from the day of Aβ1-42 peptide infusion, these rats received **N-Acetyl Cysteine Amide** (NACA), 75 mg/kg/day (ip) for seven consecutive days.
v. **N-Acetyl Cysteine Amide + Amyloid-β + N-Acetyl Cysteine Amide (N + Aβ + N) group:** The rats in this group were treated with NACA (75 mg/kg/day, ip) for seven consecutive days. This was followed by infusion of 5 µL of Aβ1-42 peptide (5 µg Aβ1-42/5 µL sterile saline) into each lateral ventricle. The NACA treatment (75 mg/kg/day, ip) was then continued further for seven more days (*n* = 18/group).(Supplementary Figure S9).

All rats in each group were subjected to cognitive functional tests. We used twelve rats in each group for biochemical studies and six rats in each group for morphological studies. After completion of NACA treatment in the Aβ, Aβ + N and N + Aβ + N groups, and saline in the NC and SH groups, rats in all groups were subjected to the Morris water maze test to test spatial learning and memory. In addition, all rats were subjected to passive avoidance tests to test avoidance learning and memory. After behavioral tests (~ten days after the last dose of NACA or saline treatment), rats were either perfused with saline followed by freshly prepared 4% paraformaldehyde (in 0.1 M phosphate buffer, pH 7.4) for histopathological studies or perfused with cold saline for collecting the fresh unfixed tissue for biochemical studies. Paraformaldehyde fixed tissues were post fixed in the same fixative for 48 h and processed for paraffin section cutting. Five-micron thick paraffin sections were stained with cresyl violet stain or immunostained with doublecortin (for neurogenesis), Synaptophysin, Aβ1-42, Tau, NeuN (for neurons), GFAP (for astrocytes) and Iba1(for microglia). Fresh unfixed tissue samples from the medial prefrontal cortex and hippocampal were dissected and instantly frozen in liquid nitrogen for biochemical studies (doublecortin, synaptophysin, amyloid-β protein by Western blot analysis and MDA, GSH and total antioxidants by ELISA analysis) and stored at −80 °C until analysis. Experimental design and timeline of the experiment are illustrated in Supplementary Figures S8 and S9 respectively.

### 4.3. Stereotaxic Surgery and Intracerebroventricular Infusion of Aβ1-42 Peptide or Normal Saline

Rats were deeply anesthetized with intramuscular injection of ketamine (80 mg/kg)-xylazine(10 mg/kg) anesthetic cocktail. The fur over the skull was trimmed using a shaving machine. The rats were placed in a stereotaxic apparatus such that the surface of the skull remained in a horizontal plane. The skin was swept with a 70% alcohol pad, followed by a subcutaneous injection with 0.2 mL of 1% lidocaine as local anesthesia. Through a midline skin incision using a sterile No-10 size surgical blade, the skull surface was exposed and then cleared from connective tissue by a dry surgical gauze. Two drill holes aiming at the left and right lateral ventricles were made on either side of the midline using a hand drill (coordinates: anteroposterior-3.00 mm behind bregma, and mediolateral 4.00 mm lateral to midline), following the Paxinos rat stereotaxic atlas [87]. Then, 5 µL of Aβ1-42 oligomer solution (1 µg/1 µL) was infused into the lateral ventricles with a 10 µL Hamilton syringe (depth coordinate 4.5 mm from skull surface). Aβ1-42 peptide (Torics Bioscience, Cat. No. 2425, Minneapolis, MN, USA) was incubated for 72 hrs at 37 °C to make Aβ1-42 oligomer. In the sham group, the surgical procedure was the same as above, however, 5 µL of saline was infused into each lateral ventricle. The skin wound was closed via absorbable coated polyglactin 910 sutures (Vicryl Rapide, Ethicon Inc., Raritan, NJ, USA), and an antiseptic betadine solution was applied to the wound before dressing. Rats were kept warm during recovery from anesthesia. An ordinary desk lamp was placed over the rats to ensure a post-operative warm environment. Each rat that underwent surgery was housed in an individual cage with a clean disposable under pad.

### 4.4. NACA Administration

N-acetylcysteine amide (NACA, Sigma-Aldrich, St. Louis, Missouri, USA, catalogue No.: A0737) was dissolved in normal saline (75 mg/mL) and administered for the rats in the Aβ, Aβ + N and N + Aβ + N groups at a dose of 75 mg/kg/day ip for the number of days mentioned in the experimental design.

### 4.5. Morris Water Maze Test

The water maze consisted of a circular water tank, 2.0 m in diameter, divided into four virtual quadrants, and a circular platform placed one centimetre under water surface in one of the four virtual quadrants, named as the platform quadrant. The temperature of the water in the tank was 20 °C, cold enough to encourage the rats to swim and reach the hidden platform to get out of the water. The walls of the experiment room were covered with cues to help the rats locate the hidden platform. The water maze test consisted of training or learning sessions and memory retention/probe tests. In learning sessions, the rats were trained in the water maze over the course of six days (one session on first day and two sessions/day from second to sixth day). Each session consisted of three trials, and each trial lasted for 120 s with an inter-trial interval of 60 s. In each trail, rats were released into water in one of the predetermined quadrants, except the platform quadrants. In each trail, total distance travelled and latency to reach the hidden platform in the platform quadrant were measured and analysed using EzVideoTM 5.70 digital video tracking system (Accuscan Instruments, Inc., Columbus, OH, USA) [88]. The memory retention test was conducted twenty-four hours after the last learning session. The memory retention test (probe test) lasted for 30 s. During this test, the platform was removed, and the rat was released in the quadrant opposite to the platform quadrant. The time spent in the platform quadrant, distance travelled in the platform quadrant, and latency to reach the platform quadrant were measured and analysed using EzVideoTM 5.70 digital video tracking system [88].

### 4.6. Passive Avoidance Test

After the Morris water maze test, rats were subjected to a passive avoidance test to test their avoidance learning and memory. The normal behaviour of rats is to explore the dark and narrow places and hide from the bright, light and wide places. The passive avoidance test tests the ability of rats to suppress this behaviour passively after receiving a noxious foot shock. In this experiment, rats were trained to identify the smaller, dark compartment of the passive avoidance apparatus during learning sessions and an electric foot shock was delivered when the rat entered the dark compartment during the last learning sessions. The normal rats remember the area where they received foot shock to avoid entering a dark compartment during a memory retention test.

The passive avoidance apparatus was made up of plexiglass and constituted of a large bright compartment (40 cm × 40 cm × 40 cm) and a small dark compartment (15 cm × 10 cm × 10 cm). The apparatus was kept in a dimly lit room where the bright compartment was illuminated with a 15 W bulb, and the dark compartment was kept relatively dark. The floor of the dark compartment was made up of stainless-steel rods spaced at equal distance, and it was connected to an electrical stimulator (10–100 V, 500 mA, direct current). Dark and bright compartments were separated from each other by a sliding door. The passive avoidance test consisted of passive avoidance learning sessions. During each passive avoidance learning session, rats were trained to explore both the bright and dark compartments for five minutes. For each rat, three trials were given with a 5-min inter-trial interval. In each learning trail, the time spent in each compartment was recorded and analysed by the Ezvideo570DV video tracking system (EzVideoTM 5.70 digital video tracking system). At the end of the third trial, rats were confined in the dark compartment (by closing the sliding door), and an electric foot shock was given for 3 s from the stimulator (75 V, 500 mA, direct current). A passive avoidance memory retention test was done twenty-four hours after the last learning session. The memory retention test, which lasted for five minutes, consisted of the rats exploring both compartments. The time spent in each compartment was recorded and analysed by the Ezvideo570DV video tracking system (EzVideoTM 5.70 Digital Video Tracking system) [89].

### 4.7. Tissue Fixation and Processing for Section Cutting

After the behavioural tests, rats were euthanized with carbon dioxide gas. The heart’s left ventricle was exposed and perfused with 100 mL of heparinized saline then with 250 mL of 4% freshly paraformaldehyde fixative. The brain was dissected (hippocampus and prefrontal cortex) and post-fixed for 48 h in a 4% paraformaldehyde fixative. Tissues were processed for paraffin section cutting. Brain tissues were dehydrated in ascending grades of ethyl alcohol, cleared in xylene and embedded in paraffin. Tissue blocks were then cut in a rotary microtome (5-μm-thick sections) and mounted on poly-L-lysine-coated glass slides. These sections were used for cresyl violet staining and several immunostainings.

### 4.8. Cresyl Violet Staining

The morphological features of the neurons in different subregions of the hippocampus and medial prefrontal cortex were studied by cresyl violet (CV) staining, which stains neurons and Nissl substances in the cytoplasm of neurons and glial cells. Paraffin sections (5 μm thick) mounted on gelatinized slides were deparaffinized in xylene (5 min) and rehydrated in descending grades of alcohol,100%, 90%, 70% and 50%, for 3 min each. The sections were stained with a 0.1% CV stain for 20 min at 60 °C. To get optimal staining, the over-stained sections were differentiated in 70% alcohol to retain optimal staining. After that, sections were dehydrated twice in 90% and absolute alcohol (5 min each). The sections were air-dried for 10 min and then cleared in two changes of xylene for 5 min each before being cover-slipped with dibutyl phthalate polystyrene xylene (DPX).

### 4.9. Immunostaining (DCX, SYN, Aβ, Tau, NeuN, GFAP and Iba1)

The brain sections were immunostained for the following proteins: doublecortin (DCX—a marker for newly generated young neurons), synaptophysin (SYN—a marker for neuronal synapses), beta-amyloid protein (Aβ—a marker for Aβ protein and plaques), tubulin-associated unit protein (Tau—a marker for Tau protein phosphorylation), neuronal nuclear protein (NeuN—a marker for mature neurons), glial fibrillary acidic protein (GFAP—a marker for astrocytes) and ionized calcium binding adaptor molecule1(Iba1—a marker for microglia). Paraffin sections were used for all immunostainings. Briefly, the sections were deparaffinized in xylene and boiled in a citrate buffer (0.1 M Sodium citrate, pH 6.4) in a microwave at medium power for 3–5 min for antigen retrieval. They were then cooled to room temperature and washed with phosphate-buffered saline (PBS, 0.1 M, pH 7.4). Sections were incubated in PBS containing 20% methanol and 3% hydrogen peroxide for 20 min at room temperature to remove endogenous peroxidase activity in the tissue. Then, the sections were washed three times (5 min each) with PBS. Next, the sections were blocked with PBS containing 10% appropriate normal serum (serum of the host animal in which a secondary antibody was developed) with 0.01% Triton X-100 for 30 min. Sections were then incubated with the appropriate primary antibodies at proper dilution overnight at 4 °C in a humidified chamber. (Rabbit anti NeuN, (1:500) (Millipore, Catalogue No.: MAB377), Mouse anti DCX, (1:200) (Santa Cruz, Catalogue No.: SC271390), Mouse anti synaptophysin (1:200) (Millipore, Catalogue No.: S5768), Mouse Anti-Tau-1 (1:500) (Millipore, Catalogue No.: MAB3420), Rabbit anti Aβ antibody (1:500) (cell signaling, Catalogue No.: D54D2), Rabbit anti GFAP (1:200) (life technologies, Catalogue No.: 180063) and Rabbit anti Iba1 (1:500) (Wako, Catalogue No.: 019-19741). Sections were washed three times with PBS. Sections were incubated with the appropriate biotinylated secondary antibodies, (Biotinylated goat anti rabbit IgG (Vector Labs, Newark, CA, USA, BA1000), or Biotinylated goat anti-mouse IgG, (Vector Labs, BA9200) then diluted at 1:200 in 3% normal serum for one hour at room temperature in a humid chamber. Sections were then washed three times with PBS and incubated with avidin–biotin complex for one hour at room temperature in a humid chamber. Sections were then colour developed with diaminobenzidine (vector grey for DCX) as a chromogen. The sections were counter-stained with haematoxylin, dehydrated in ascending grades of ethyl alcohol, cleared with xylene and mounted using DPX mounting media.

### 4.10. Western Blotting for DCX, Aβ and SYN

For Western blot analysis, tissue samples which were collected and stored at −80 °C were used. For sample preparation, tissue samples were thawed to 4 °C and homogenized in a known volume of radioimmunoprecipitation assay buffer [RIPA buffer: 500 mL of the buffer contains 50 mM Tris, pH 7.4, 3.94 g + 150 mM Na Clb 4.38 g + 1% NP—40 (Sigma Chemicals, Perth, WA, USA) 5 mL + 5 mM EDTA 0.73 g + 0.1% SDS + protease inhibitors (Roche) 100 µL/mL buffer + 2 mM benzamidine (Fluka) 0.12 g + phenylmethyl sulfonyl fluoride (PMSF: Sigma Chemicals) 10 µL/mL and 0.5% Na-deoxycholate (Sigma Chemicals)]. Homogenate was centrifuged at 14,000× *g* for 20 min in a refrigerated (4 °C) centrifuge. The protein level in the sample was measured by the Bradford method. Samples were denatured for 5 min in a boiling water bath. The soluble protein fractions in the sample were separated by electrophoresis in a 10% SDS-polyacrylamide gel (30 µg protein/well) and electroblotted to a polyvinylidene fluoride membrane (PVDF: Millipore, Burlington, MA, USA) in a blotting apparatus (Bio-Rad Laboratories, Inc., Hercules, CA, USA). The membrane was then blocked with 5% dried skim milk, TRIS-buffered saline (TBS, 10X), with 0.5% Tween 20 at room temperature for 1.5–2 h and incubated with appropriately diluted primary antibodies overnight at 4 °C on a shaker [(Goat anti DCX (Sigma-Aldrich, SAB2501666), Mouse anti synaptophysin (Sigma-Aldrich, Catalogue No.: MAB5258), Rabbit anti Aβ1-42 polyclonal antibody (Abcam, Catalogue No.: ab10148)]. After washing the membrane three times with TRIS-buffered saline (TBS, 1X), with 0.5% Tween 20, incubated it for two hours at room temperature with appropriate diluted horseradish peroxidase-conjugated secondary antibodies [(Horse anti-goat IgG H&L (Abcam, Catalogue No.: ab205718), Goat Anti-mouse IgG H&L (Abcam, Catalogue No.: ab205718), Goat Anti-rabbit IgG H&L (Abcam, Catalogue No.: ab205718)], and then rewashed once with TRIS-buffered saline (TBS, 1X), with 0.5% Tween 20. The polyvinylidene fluoride membrane was treated with enhanced chemiluminescent substrate and exposed to an X-ray hyper-film. Using antibodies specific for β-actin, the same blots were re-incubated to confirm equal protein loading (Abcam, UK; 1:1000). Protein band densities were measured using a calibrated densitometer and normalized to the respective β-actin values.

### 4.11. Analysis of Oxidant/Antioxidants in Hippocampal and Medial Prefrontal Cortex Tissue ELISA Method

For antioxidant studies, tissue samples, which were collected stored at −80 °C, were used. The tissues were thawed and soaked in cold saline and rinsed with saline phosphate buffer (0.1 M, pH 7.4). After that, the tissue was weighed and homogenized in 0.1 M/l saline phosphate buffer (1:10 *w*/*v*). Finally, the homogenate was centrifuged at 10,000 γ for 20 min at 4 °C.

#### 4.11.1. Estimation of Malondialdehyde (MDA)

Lipid peroxidation in the tissue was measured as described by Ohkawa et al. [90], using a lipid peroxidation kit (myBioSource, Cat.no-MBS2540553). Using hippocampal or medial prefrontal cortical tissue homogenate at 37 °C for 30 min, lipid peroxidation was measured. After centrifugation, the supernatant (1 mL) was mixed with 1 mL of 0.65% thiobarbituric acid. The mixture was then kept in a boiling water bath for 15 min. The MDA formation was determined by reading absorbance at 535 nm. Thiobarbituric acid reactive substances (TBARS) were calculated using a molar extinction coefficient of 1.56 × 105 (M) − 1 cm^−1^.

#### 4.11.2. Estimation of Reduced GSH

A method described by Ellman [91] and Bernhardt et al. [92] was used to estimate GSH levels in the brain with a reduced glutathione kit (myBioSource, Cat.no-MBS046356). First, we precipitated a 1 mL supernatant with 1 mL of 4% sulfosalicylic acid and cold digested it for one hour at 41 °C. Then, at 1200 revolution per minute, samples were centrifuged for 15 min and 1 mL of the supernatant was added to 2.7 mL of phosphate buffer at pH 8 and 0.2 mL of 2,50-dithio-bis (2-nitrobenzoic acid). Immediately after that, the developed yellow colour was read at 412 nm using the UV-1700 spectrophotometer (Shimadzu, Japan). Using the molar extinction coefficient of the chromophore (1.36 × 104/mol/L/cm), results were calculated and expressed as nmol per mg protein.

#### 4.11.3. Estimation of Total Antioxidants

Based on Koracevic et al. [93] and Bernhardt et al. [92], the total antioxidant capacity of the brain tissue was assessed with the total antioxidants (myBioSource, Cat.no.MBS2540515). This method measured the ability to remove hydrogen peroxide when added. Using an enzyme reaction, we determine the remaining hydrogen peroxide colorimetrically by measuring the colour at 532 nm of a product resulting from the conversion of 3-dichloro-2-hydroxyl benzenesulfonate.

#### 4.11.4. Statistical Analysis

All data were expressed as mean ± standard error of mean (SEM) and analysed using the SPSS statistical analysis software (version 25). Data analysis was done using a two-way analysis of variance (ANOVA, repeated measures) test (Morris water maze data) or a one-way ANOVA (all other data) followed by Bonferroni’s multiple comparison post hoc test. *p*-values < 0.05 were considered as significant.

## Data Availability

The data that support the findings of this study are available from the corresponding author upon reasonable request.

## Supplementary Materials

The supporting information can be downloaded at: https://www.mdpi.com/article/10.3390/ijms241612733/s1.
