# Correction: Alkandari et al. N-Acetylcysteine Amide Against Aβ-Induced Alzheimer’s-like Pathology in Rats. *Int. J. Mol. Sci.* 2023, *24*, 12733

**DOI:** 10.3390/ijms27135898

**Published:** 2026-06-30

**Authors:** Ahmed Fareed Alkandari, Sampath Madhyastha, Muddanna S. Rao

**Affiliations:** Department of Anatomy, College of Medicine, Kuwait University, P.O. Box 24923, Safat 13110, Kuwait; ahmed.adbullah@grad.ku.edu.kw (A.F.A.); muddanna.rao@ku.edu.kw (M.S.R.)

In the original publication [1], there was an error in Figure 10 (Tau protein expression), two images were identical (Aβ+N and N+Aβ+N) for dentate gyrus and CA1 regions (first and second columns). It was an error as published. The corrected Figure 10 Aβ+N for dentate gyrus (DG) in the first column and Aβ+N for CA1 in the second column are replaced with correct images. The authors state that the scientific conclusions are unaffected. This correction was approved by the Academic Editor. The original publication has also been updated.


