# Supplementary material for: N-Acetylcysteine Amide against Aβ-Induced Alzheimer’s-like Pathology in Rats
Source: Int J Mol Sci. 2023 Aug 12;24(16):12733. doi: 10.3390/ijms241612733 (PMC10454451; doi:10.3390/ijms241612733)
Supplement: Supplementary file 1 [file ijms-24-12733-s001.zip › ijms-2460917-supplementary.pdf]

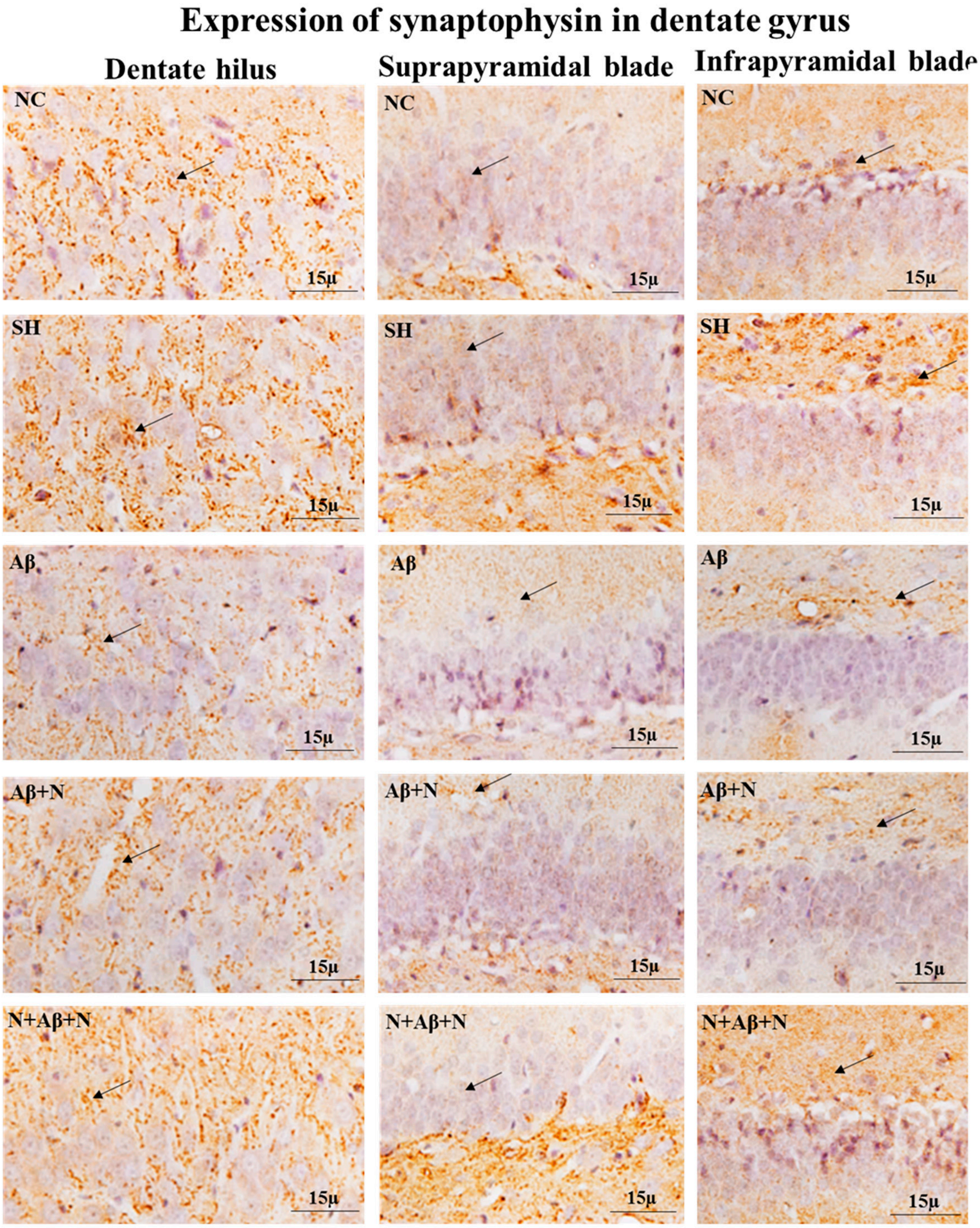

Fig.S1: Synaptophysin expression in the dentate hilus, supra and infrapyramidal blade regions in the various groups demonstrated by immunostaining the hippocampal sections with anti-SYN antibody. Note the diminished SYN expression (arrow) in Aβ group compared to NC/SH groups, and the increased expression in Aβ+N and N+Aβ+N groups compared to the Aβ group.

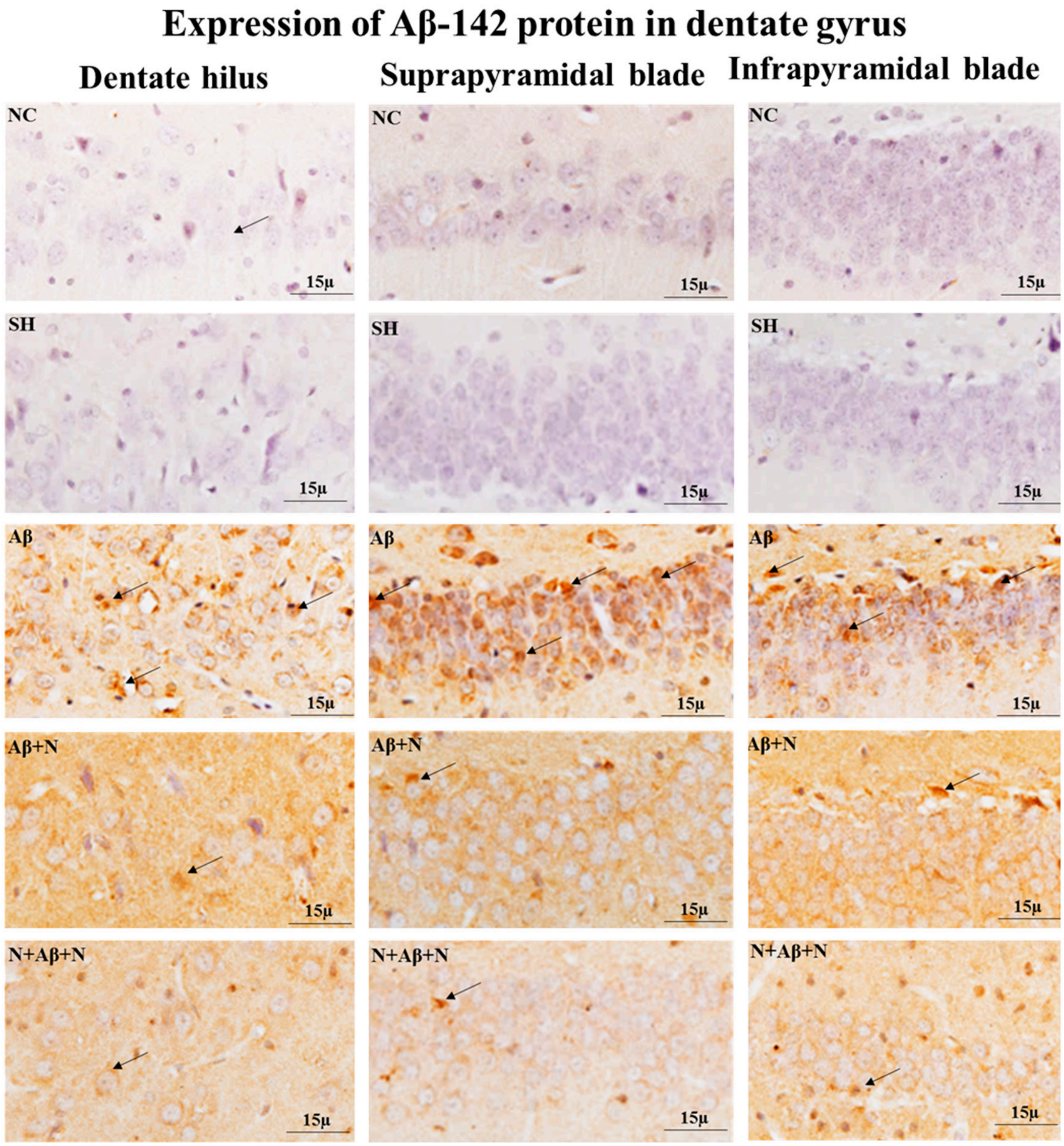

Fig.S2: Aβ protein expression in the dentate hilus, supra and infrapyramidal blade regions in the various groups demonstrated by immunostaining the sections with anti-Aβ antibody. Note the increased expression of Aβ in the Aβ group compared to NC/SH groups, and the decreased expression in Aβ+N and N+Aβ+N groups compared to Aβ group.

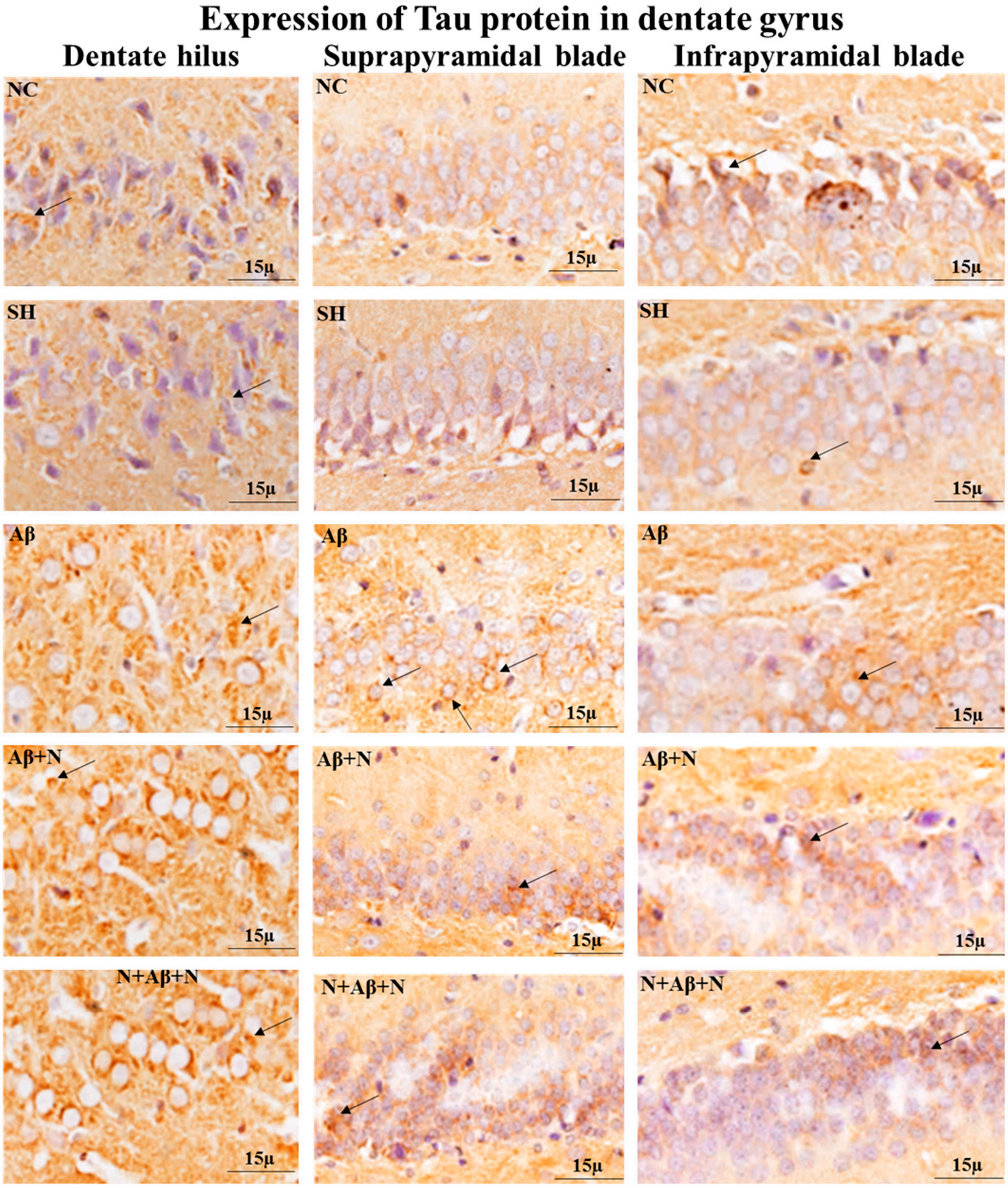

Fig.S3: Tau expression in the dentate hilus, supra and infrapyramidal blade regions in the various groups demonstrated by immunostaining the sections with anti-Tau antibody. Note the increased expression of Tau in the Aβ group compared to NC/SH groups, and the decreased expression in Aβ+N and N+Aβ+N groups compared to Aβ group.

Cresyl Violet staining – Dentate gyrus

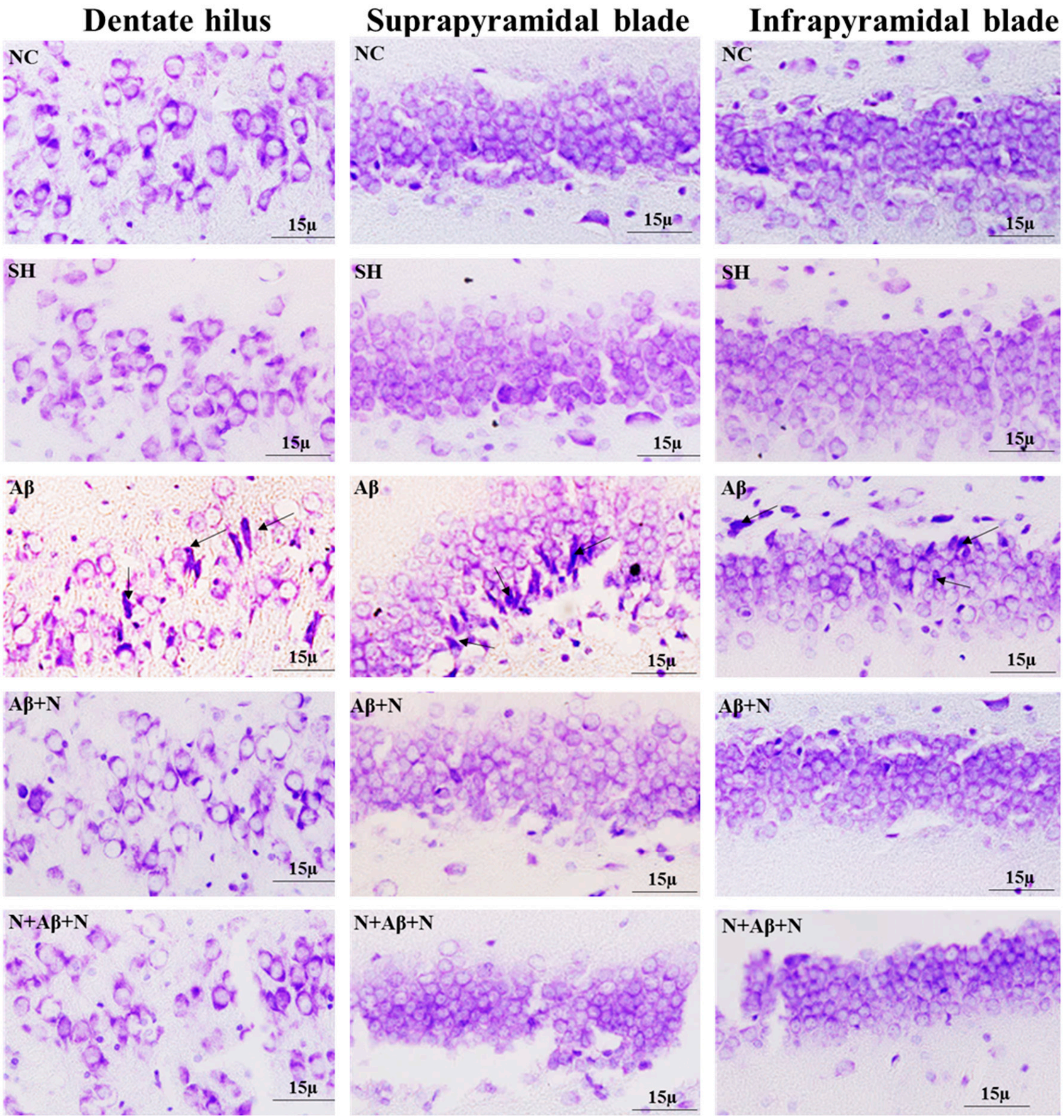

Fig.S4: Cresyl violet staining of the dentate hilus, supra and infrapyramidal blade regions in the various groups. Note the degenerated neurons (arrow) in the Aβ group.

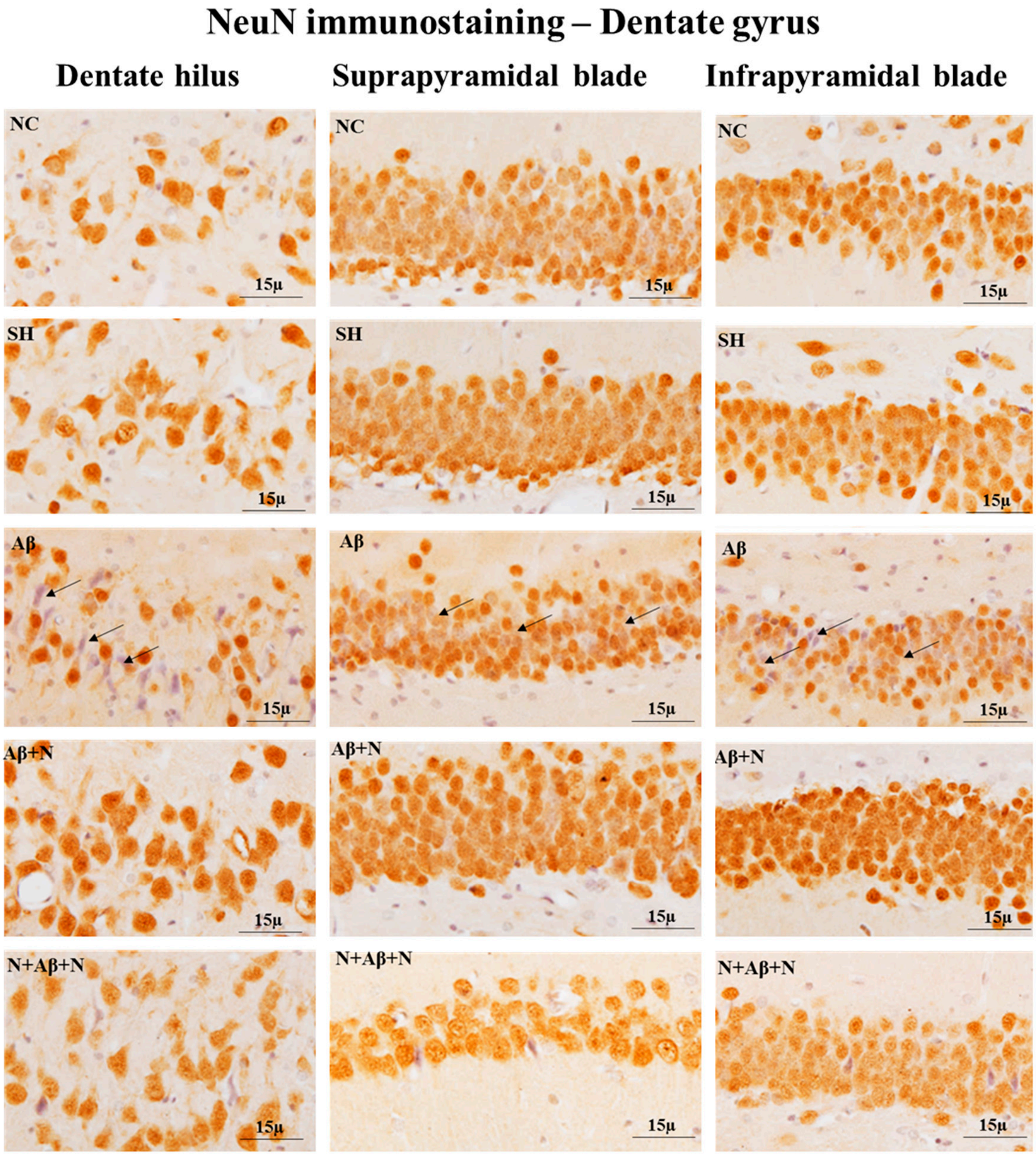

Fig.S5: NeuN immunostaining in the dentate hilus, supra and infrapyramidal blade regions in the various groups. Note the diminished mature neurons in the Aβ group.

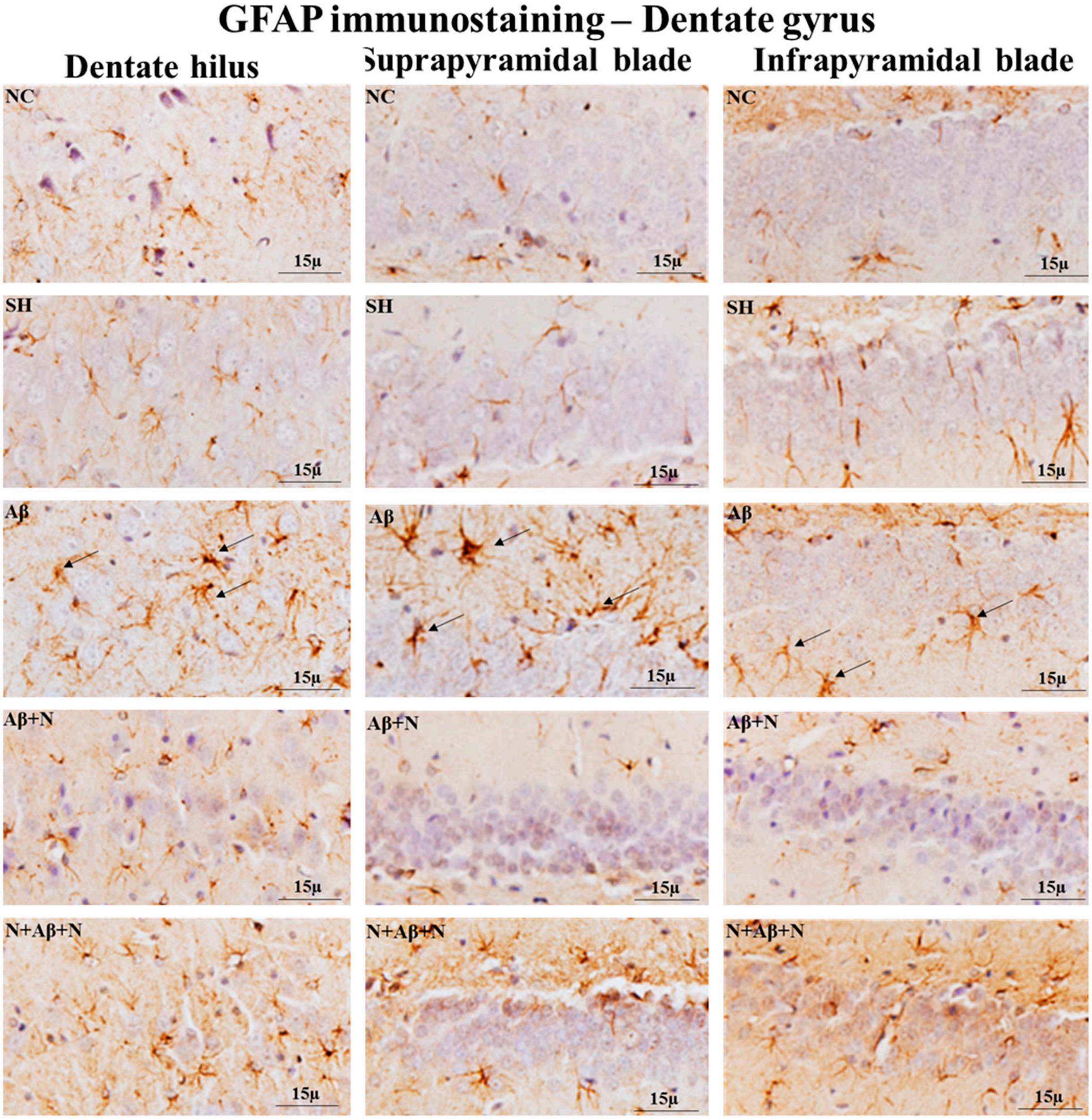

Fig.S6: GFAP immunostaining for astrocytes in the dentate hilus, supra and infrapyramidal blade regions in the various groups. Note the elevated number of astrocytes (arrow) in Aβ group.

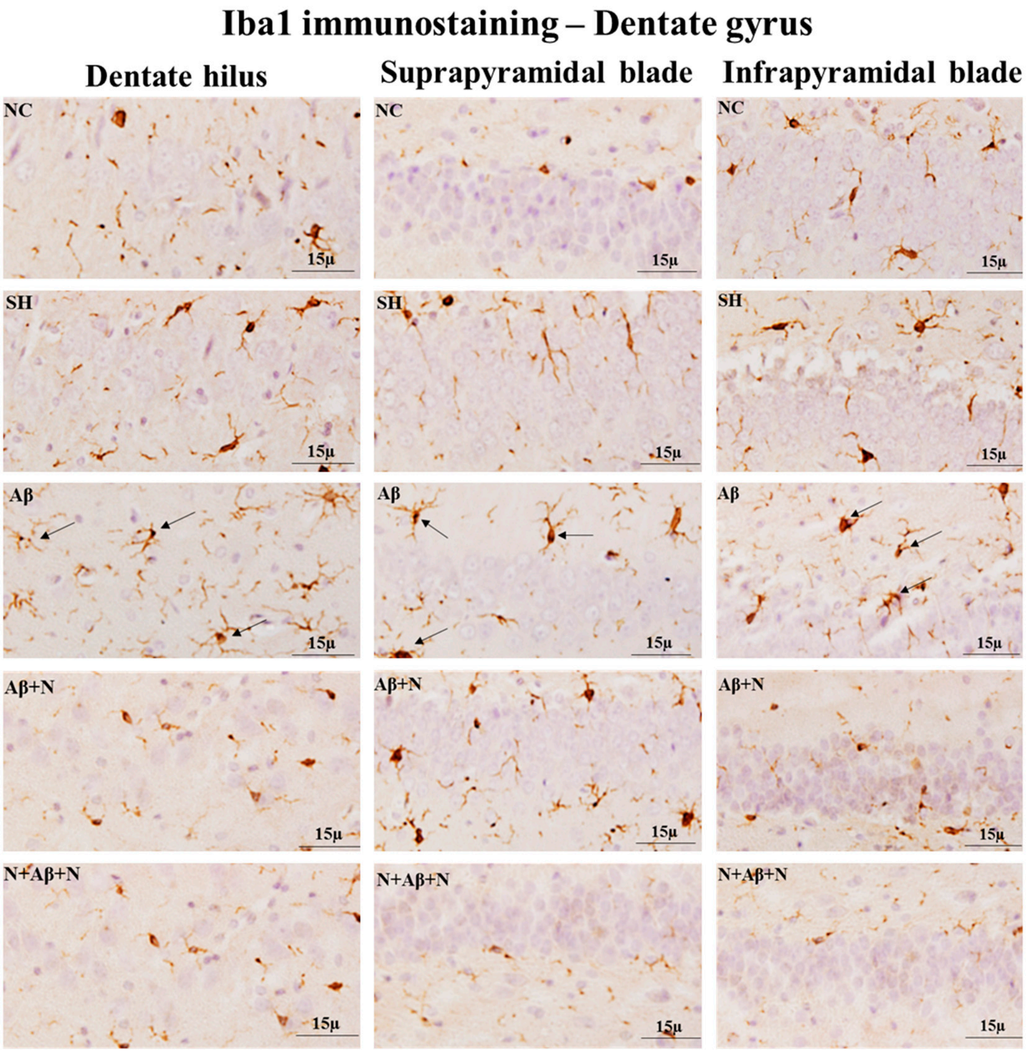

Fig.S7: Iba1 immunostaining for microglia in the dentate hilus, supra and infrapyramidal blade regions in the various groups. Note the increased microglia in the Aβ group.

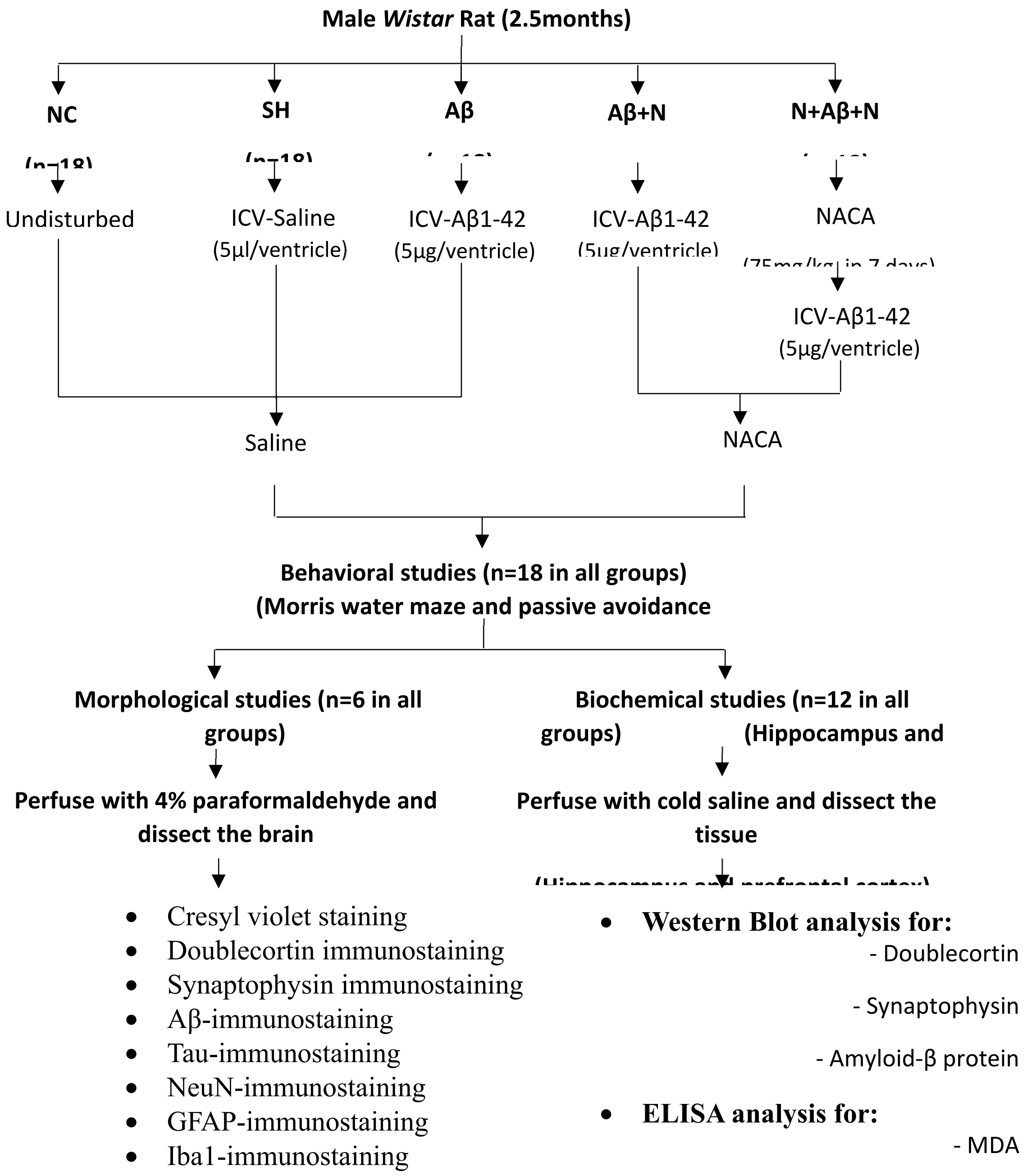

Fig.S8. A schematic flow chart showing experimental design and animal groups

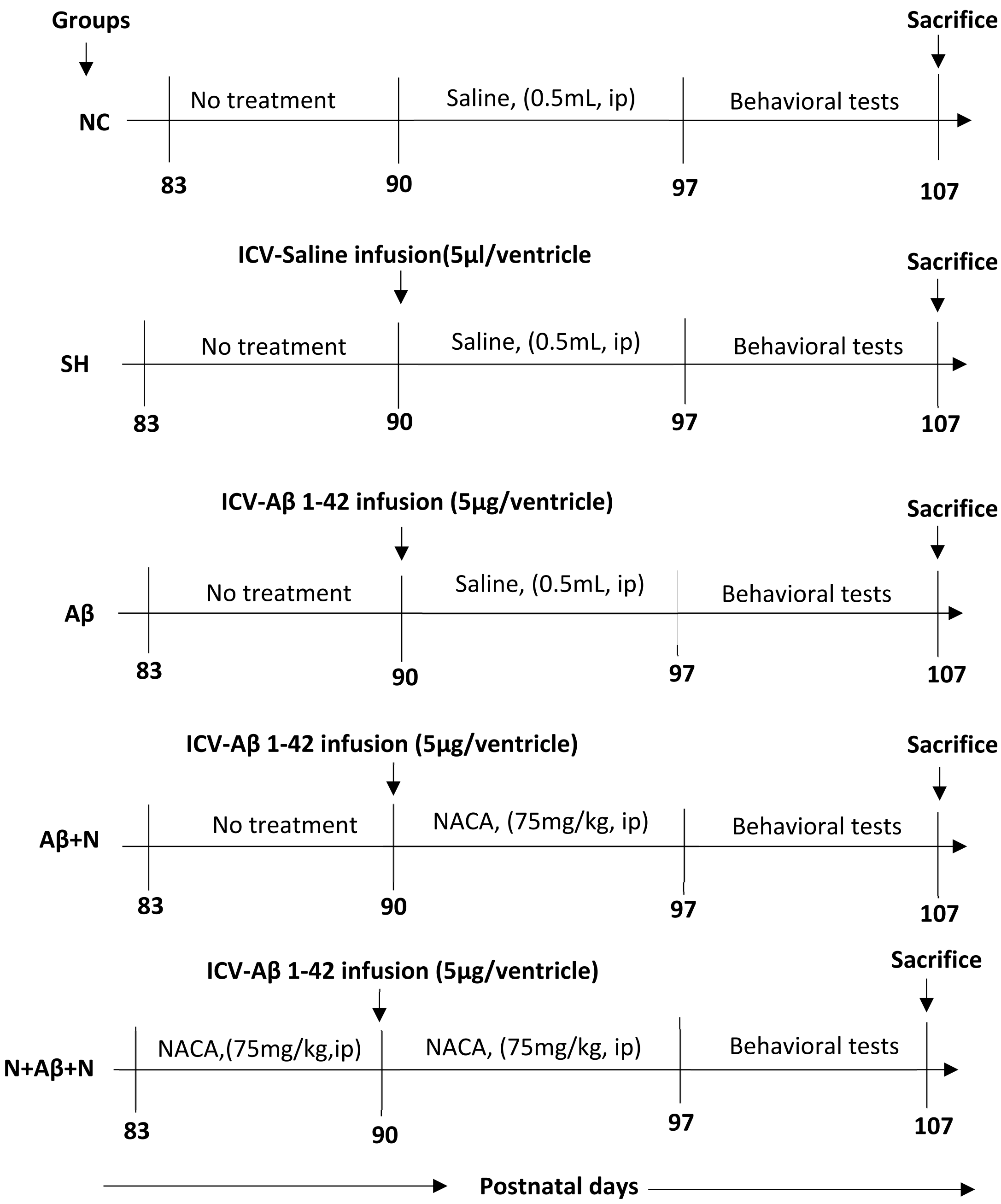

Fig.S9. A schematic illustration showing timelines of experiment
